# Carrier-free mRNA vaccine induces robust immunity against SARS-CoV-2 in mice and non-human primates without systemic reactogenicity

**DOI:** 10.1016/j.ymthe.2024.03.022

**Published:** 2024-04-02

**Authors:** Saed Abbasi, Miki Matsui-Masai, Fumihiko Yasui, Akimasa Hayashi, Theofilus A. Tockary, Yuki Mochida, Shiro Akinaga, Michinori Kohara, Kazunori Kataoka, Satoshi Uchida

**Affiliations:** 1 Innovation Center of NanoMedicine (iCONM), Kawasaki Institute of Industrial Promotion, 3-25-14 Tonomachi, Kawasaki-ku, Kawasaki 210-0821, Japan; 2 Department of Research, NANO MRNA Co., Ltd., 3-25-14 Tonomachi, Kawasaki-ku, Kawasaki 210-0821, Japan; 3 Department of Diseases and Infection, Tokyo Metropolitan Institute of Medical Science, 2-1-6, Kamikitazawa, Setagaya-ku, Tokyo 156-8506, Japan; 4 Department of Pathology, Kyorin University School of Medicine, 6-20-2 Shinkawa, Mitaka-shi, Tokyo 181-8611, Japan; 5 Department of Advanced Nanomedical Engineering, Medical Research Institute, Tokyo Medical and Dental University (TMDU), 1-5-45 Yushima, Bunkyo-ku, Tokyo 113-8510, Japan; 6 Department of Microbiology and Cell Biology, Tokyo Metropolitan Institute of Medical Science, 2-1-6, Kamikitazawa, Setagaya-ku, Tokyo 156-8506, Japan

**Keywords:** mRNA vaccine, COVID-19, mRNA delivery, infectious disease vaccine, reactogenicity, non-human primate, jet injector, lipid nanoparticle, adjuvant

## Abstract

Carrier-free naked mRNA vaccines may reduce the reactogenicity associated with delivery carriers; however, their effectiveness against infectious diseases has been suboptimal. To boost efficacy, we targeted the skin layer rich in antigen-presenting cells (APCs) and utilized a jet injector. The jet injection efficiently introduced naked mRNA into skin cells, including APCs in mice. Further analyses indicated that APCs, after taking up antigen mRNA in the skin, migrated to the lymph nodes (LNs) for antigen presentation. Additionally, the jet injection provoked localized lymphocyte infiltration in the skin, serving as a physical adjuvant for vaccination. Without a delivery carrier, our approach confined mRNA distribution to the injection site, preventing systemic mRNA leakage and associated systemic proinflammatory reactions. In mouse vaccination, the naked mRNA jet injection elicited robust antigen-specific antibody production over 6 months, along with germinal center formation in LNs and the induction of both CD4- and CD8-positive T cells. By targeting the SARS-CoV-2 spike protein, this approach provided protection against viral challenge. Furthermore, our approach generated neutralizing antibodies against SARS-CoV-2 in non-human primates at levels comparable to those observed in mice. In conclusion, our approach offers a safe and effective option for mRNA vaccines targeting infectious diseases.

## Graphical abstract

**Figure:**
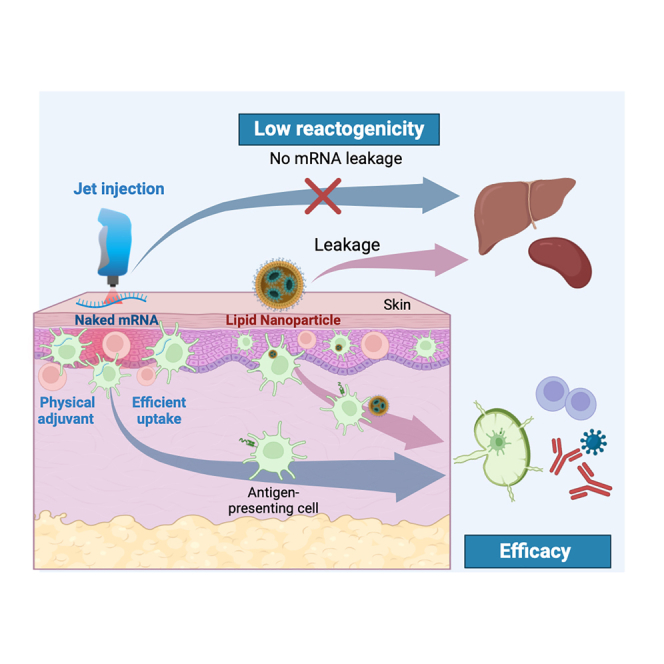

## Teaser

Uchida and colleagues developed carrier-free mRNA vaccines using a jet injector that targets dermal tissues. The vaccine elicited a robust immune response against SARS-CoV-2 in mice and non-human primates. This approach confined the vaccine distribution to the injection site, preventing any systemic mRNA leakage and the resulting systemic proinflammatory reactions.

## Introduction

The use of antigen-encoding messenger RNA (mRNA) has instigated a paradigm shift in vaccine development because of its high efficiency in vaccination, ease of mRNA sequence designing, and scalability for billions of doses per year.^1^^,^^2^^,^^3^ In 2020, two novel mRNA vaccines, BNT162b2 and mRNA-1273, received emergency approval for use against the coronavirus disease 2019 (COVID-19) caused by SARS-CoV-2 infection, showing >90% efficacy in preventing the symptomatic disease.^4^^,^^5^ Both vaccines use lipid nanoparticles (LNPs) as the delivery system, which protect mRNA from enzymatic degradation, facilitate its intracellular delivery, and act as an immunostimulatory adjuvant.^6^^,^^7^^,^^8^ LNPs can distribute broadly to the lymph nodes (LNs), spleen, and liver following local injection in small and large mammals.^8^^,^^9^^,^^10^^,^^11^^,^^12^ While the distribution of LNPs to the lymphoid organs, e.g., the spleen and LNs, benefits vaccination processes, their systemic distribution could be reactogenic. Even though mRNA-LNP vaccines have shown high safety profiles with minimal occurrence of severe adverse effects in clinical trials and real-world evidence,^4^^,^^5^^,^^13^ there is room for further reducing reactogenicity to enhance the overall benefits of mRNA vaccines. This challenge has spurred extensive research in the field to explore alternative solutions aimed at mitigating the systemic reactogenicity.^14^^,^^15^ Notably, reducing the incidence of reactogenic events could reduce vaccine hesitancy.^16^ In this context, enabling naked mRNA vaccines could enhance vaccine tolerability, establishing a new class of effective COVID-19 vaccines. However, this approach is challenging, given the multifunctional attributes of LNPs in potentiating mRNA vaccines. Indeed, the robustness of naked mRNA in protection against infectious diseases has not been well demonstrated, except for promising outcomes observed with naked self-replicating mRNA following *in vivo* electroporation.^17^^,^^18^

Optimizing the delivery route can improve vaccination efficiency. Both BNT162b2 and mRNA-1273 are administered by intramuscular (i.m.) injections. Nevertheless, the skin represents a potent vaccination target because the epidermis and dermis have higher antigen-presenting cell (APC) density than the muscle tissue.^19^^,^^20^ Recent clinical trials showed that the intradermal (i.d.) delivery of BNT162b2 and mRNA-1273 achieved dose sparing at one-tenth to one-fifth of the i.m. dose, with a trend of alleviated adverse effects.^21^^,^^22^^,^^23^ However, administering i.d. vaccines requires highly trained medical personnel, making it difficult to perform routinely in the clinic.^24^

Besides the difficulty in the procedure of i.d. administration, naked mRNA as i.d. vaccines remains challenging because of the poor stability and inefficient cellular uptake of mRNA in the skin.^25^ Indeed, naked mRNA i.d. vaccines yielded little or no antibody responses and only modest CD8+ cellular immunity previously.^18^^,^^26^^,^^27^ The rapid delivery kinetics are crucial for internalizing naked mRNA into the cells before its degradation in the cutaneous space. To address these issues, herein, we employed a needle-free, pyro-drive liquid jet injector (PYRO), which facilitates the internalization of macromolecules into skin cells through the instantaneous rise of liquid pressure without any complicated administration procedure.^28^^,^^29^ As a result, rapid cellular uptake of mRNA following PYRO injection allowed for overcoming enzymatic mRNA degradation, leading to efficient protein production from naked mRNA in the skin. Moreover, we revealed a potential function of PYRO injection as an immunostimulatory adjuvant. PYRO injection elicited localized proinflammatory reactions at the injection site, presumably by provoking physical stress at the injection site. Such proinflammatory reactions may serve as a physical adjuvant to potentiate immune responses. From a safety viewpoint, our approach of using naked mRNA restricted mRNA distribution to the injection site, avoiding systemic proinflammatory reactions after vaccination. Ultimately, PYRO-injected naked mRNA elicited robust vaccination effects in mice and non-human primates (NHPs) without systemic reactogenicity and provided a protective effect in a SARS-CoV-2 challenge test in mice.

## Results

### PYRO boosts the delivery and vaccination efficiency of naked mRNA

**Figure 1:**
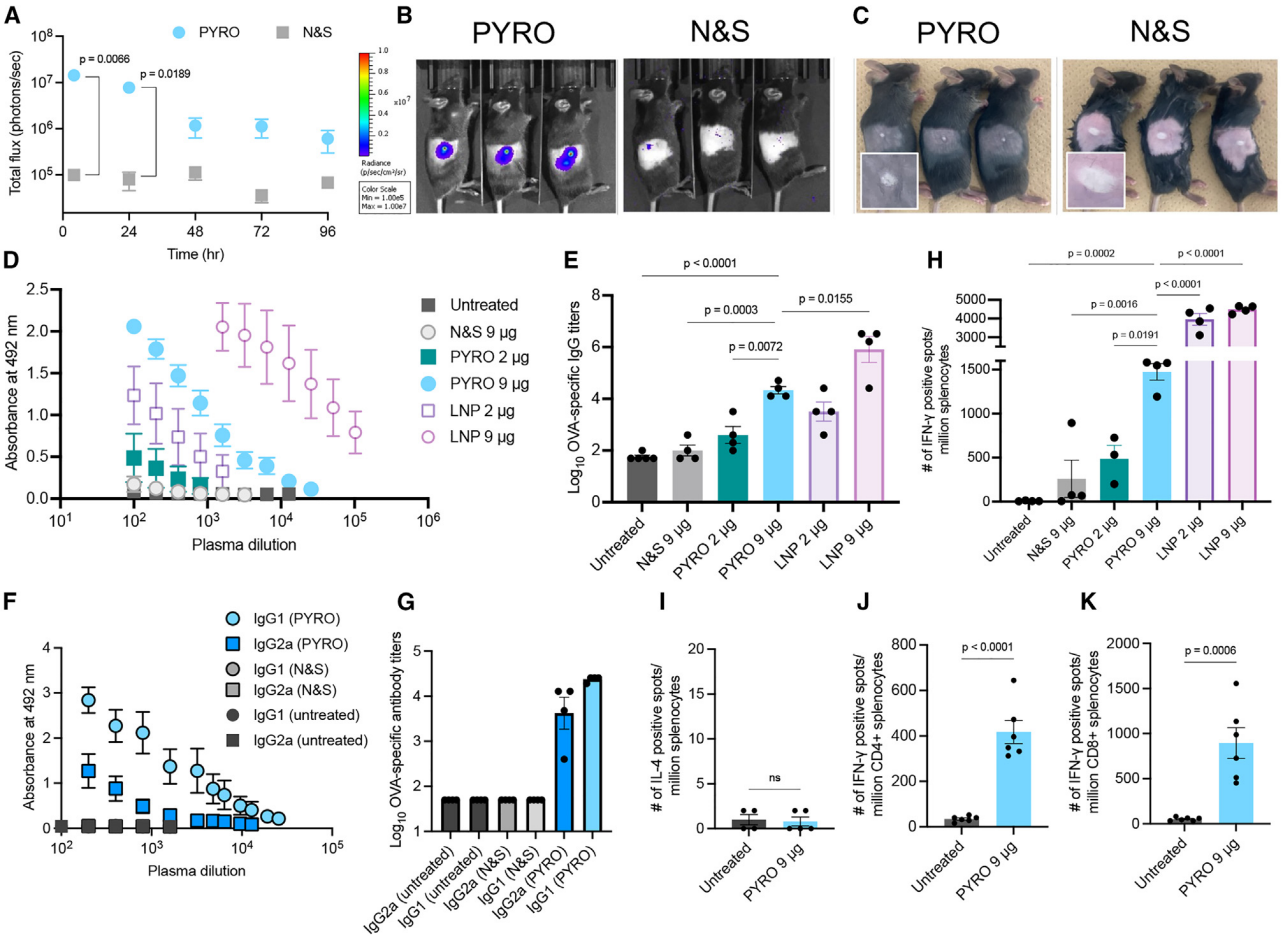
The efficiency of mRNA delivery and anti-OVA vaccination (A–C) Naked mRNA encoding luciferase was i.d. injected into the right flank of mice. (A) Luciferase expression levels quantified by IVIS imaging. n = 3. (B) Representative IVIS-acquired images showing luciferase expression around the injection site. (C) The appearance of the injection site after i.d. injection of naked mRNA using N&S and PYRO. (D–K) Mice were immunized with *OVA* mRNA twice at a 3-week interval, and blood and spleens were collected 2 weeks after the second dose. (D and E) OVA-specific total IgG. (F and G) OVA-specific IgG isotypes. (D and F) Absorbance vs. dilution curves. n = 4. (E and G) Log-transformed titers. n = 4. (H and I) Cellular immunity as measured by IFN-γ ELISpot (H), and IL-4 ELISpot (I). n = 4. (J and K) Number of OVA-reactive IFN-γ-producing CD4+ and CD8+ T cells in the spleens of vaccinated mice. n = 6. Data represent the mean ± SEM. Statistical analyses were performed by one-way ANOVA followed by Tukey’s post hoc test in (E) and (H) and unpaired Student’s t test in (A), (I), (J), and (K). n.s., nonsignificant.

Prior to conducting *in vivo* experiments, using a capillary electrophoresis, we confirmed that physical stress during the jet injection process caused minimal mRNA degradation (Figure S1). Then, *firefly luciferase* (*fLuc*)-encoding mRNA, dissolved in 20 μL of solution, was intradermally injected to evaluate the protein expression level in the skin (Figures 1A and 1B). PYRO injection induced approximately 200-fold more efficient fLuc expression at the injection site compared with manual injection with a needle and a syringe (N&S) after 4 h of injection. The fLuc expression remained high and gradually reduced in intensity over 4 days (Figure 1A). Despite the large difference in the protein expression efficiency, PYRO and N&S injections had similar physical appearances on the skin, showing a round, whitish bleb on the surface (Figure 1C). In detail, N&S injection provided a larger bleb than PYRO injection (Table S1). The skin bleb shrank rapidly and became almost invisible within several hours (Figure S2). Histologically, injected solution primarily dispersed in dermal and subcutaneous tissues in both PYRO and N&S injections (Figure S3). The comparable tissue distribution profiles observed in these two groups allowed us to evaluate the effect of jet injection in vaccination by excluding the influence of tissue distribution.

We then evaluated the utility of PYRO in vaccination using naked mRNA encoding *ovalbumin* (*OVA*) as a model antigen. Mice received a prime and a boost separated by a 3-week interval, each consisting of 2 or 9 μg naked *OVA* mRNA dissolved in 20 μL solution. Note that the typical mRNA dose used in mouse vaccination falls within the range of 1–10 μg.^27^^,^^30^^,^^31^ As a control, we performed intradermal N&S injection of LNP based on the ionizable lipid D-Lin-MC3-DMA, a widely used control in mRNA vaccination studies.^10^ PYRO injection of naked mRNA successfully produced OVA-specific immunoglobulin (Ig)G dose-dependently, with a high titer observed at 9 μg (Figures 1D and 1E). The antibody production was almost undetectable after N&S injection of 9 μg naked *OVA* mRNA. We further evaluated the isotypes of IgG produced at the 9-μg dose group. PYRO injection produced high titers of OVA-specific IgG1 and IgG2a, which were below the detection limit in the N&S group (Figures 1F and 1G). Notably, antigen-binding IgG1 and IgG2a are both needed for protection against viral infection.^32^

The cellular immune responses of PYRO-injected naked *OVA* mRNA were also evaluated using enzyme-linked immunospot assay (ELISpot). In contrast to the absence of humoral immunity in N&S-injected mice (Figures 1D and 1E), N&S injection of naked mRNA (9 μg) induced a detectable level of OVA-specific interferon (IFN)-γ-producing splenocytes in vaccinated mice (Figure 1H), which is consistent with previous reports.^18^^,^^26^^,^^27^^,^^33^ Notably, PYRO injection of naked mRNA induced a 5-fold increase in the spot number compared with N&S injection at the dose of 9 μg (Figure 1H). As in humoral immunity, the 9-μg dose induced higher cellular immunity than the 2-μg dose. Note that N&S-injected LNP exhibited a significant enhancement of OVA-specific humoral and cellular immunity compared with PYRO injection of naked mRNA (Figures 1D, 1E, and 1H).

The splenocytes of mice injected with PYRO did not produce interleukin (IL)-4, a T helper 2 (Th2)-related cytokine (Figure 1I), but produced high levels of IFN-γ, a T helper 1 (Th1)-related molecule (Figure 1H). The production of both IgG1 and IgG2a and the lack of Th2 cytokines in the presence of Th1 cytokines indicate favorable humoral and cellular immune responses induced by PYRO injection of naked mRNA. Furthermore, the spleens of vaccinated mice possessed both helper CD4+ and cytotoxic CD8+ T cells reactive to OVA (Figures 1J and 1K). N&S injection at a 9-μg dose also produced both helper CD4+ and cytotoxic CD8+ T cells, albeit to a lesser extent than PYRO injection (Figures S4A and S4B), with a minimal number of IL-4 positive spots in an ELISpot assay using whole splenocytes (Figure S4C).

### PYRO-injected naked mRNA vaccine produces robust immunity and protection against the SARS-CoV-2 challenge in mice

**Figure 2:**
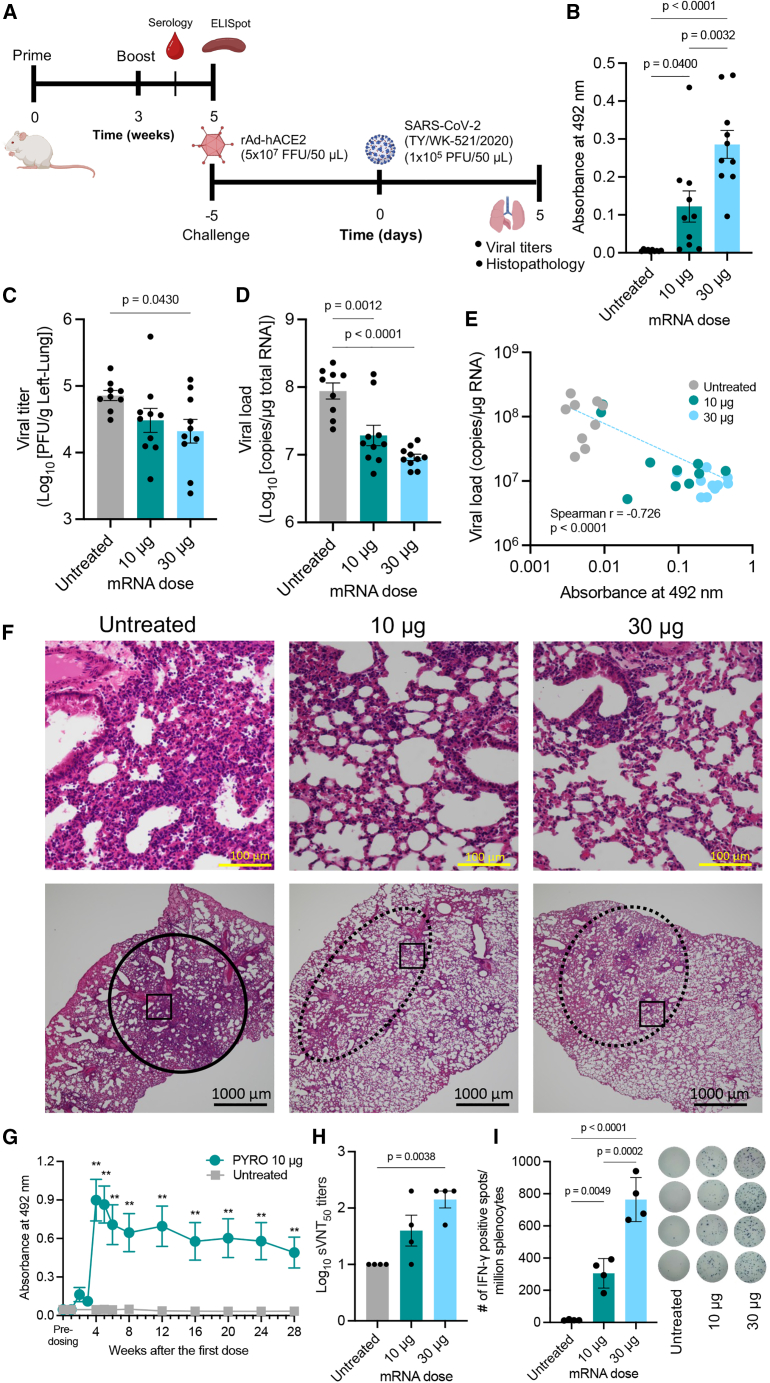
Mouse vaccination with *spike* mRNA and viral challenge (A) Immunization and viral challenge schedule. (B) Spike-specific IgG ELISA absorbance at 1,000× serum dilution following PYRO injection of naked *spike* mRNA. (C and D) Viral challenge experiment with and without PYRO immunization with naked *spike* mRNA. Log-transformed SARS-CoV-2 viral loads in the lungs of the infected mice were measured based on viral RNA amount evaluated by viral infectious activity (PFU) (C) and qPCR (D). (E) Correlation between plasma IgG levels (B) and viral RNA amount in lungs (D). n = 10 except for the untreated group (n = 9). In the untreated group, among 10 infected mice, one mouse died before the evaluation day. (F) H&E staining of the lung tissue sections. The upper figures show the magnification of the rectangle in the lower figures. A solid circle in the untreated group shows diffusive and severe inflammation. Dotted circles in vaccinated groups show the area with modest inflammation. (G) Time-dependent profile of spike-specific IgG following PYRO injection of naked *spike* mRNA at the dose of 10 μg/dose. (H) sVNT measurement of log-transformed plasma titer of vaccinated mice required for 50% inhibition of binding between ACE2 and SARS-CoV-2 RBD. n = 4. (I) ELISpot for spike-reactive IFN-γ splenocytes in vaccinated mice. The right image shows the appearance of the ELISpot plate. n = 4. Data represent the mean ± SEM. Statistical analyses were performed by one-way ANOVA followed by Tukey’s post hoc test in all figures except (C), which was followed by Dunnett’s test.

For SARS-CoV-2 mRNA vaccine development, mice were PYRO-injected with naked s*pike* mRNA at two different doses (10 and 30 μg) in a prime-boost schedule with a 3-week interval, as illustrated in Figure 2A. In this experiment, we did not include the N&S naked mRNA control, because of its negligible antibody production and low cellular immunity observed in *OVA* mRNA vaccination (Figure 1). The vaccinated mice from the two dose groups successfully produced anti-spike-IgG 1 week after the boost, with the 30-μg group showing a higher IgG level than the 10-μg group (Figure 2B). Two weeks after the boost, mice were transduced with adenoviral vectors encoding a copy of the human *angiotensin-converting enzyme 2* (*hACE2*) receptor by intranasal administration.^34^ Five days after the transduction, the mice were challenged with an early circulating strain of SARS-CoV-2. On day 5 after the challenge, we measured the SARS-CoV-2 viral load in the lung based on viral RNA amount evaluated by viral infectious activity (plaque-forming unit, PFU) (Figure 2C) and quantitative PCR (qPCR) (Figure 2D). Compared with the untreated group, vaccination significantly reduced infectious activity in the 30-μg group (Figure 2C) and viral mRNA amount in the two dose groups (Figure 2D). The 30-μg naked mRNA group achieved around 10 times reduction in viral RNA copies in the lung. The efficacy is comparable to the outcome reported in a previous study, where 1 μg mRNA-LNP was delivered i.m. to a similar mouse model prepared by viral transduction of *hACE2*.^35^ Meanwhile, several other reports have shown nearly complete protection against SARS-CoV-2 infection in animals using different animal models.^36^^,^^37^ Figure 2E plots the viral load (Figure 2D) against IgG amount (Figure 2B) in each mouse, revealing an inverse relationship between antibody production and viral loads in mice. This plot establishes that IgG produced by PYRO injection of naked mRNA has a concentration-dependent efficacy in limiting SARS-CoV-2 infection in mice. The tissue sections illustrate the reduction in lung inflammation post-vaccination (Figure 2F). In the lower-magnification images (bottom images of Figure 2F), the lung of the unvaccinated mouse exhibited noticeable alveolar collapse, particularly in the encircled area, leading to an overall decrease in air content throughout the lung. In contrast, the vaccinated mice from both dose groups showed modest alveolar collapse, confined to areas marked with dotted circles. As a result, the mice maintained higher air contents than unvaccinated mice. High-magnification images (top images of Figure 2F) supported this observation. In the unvaccinated mouse, alveoli collapse due to extensive immune cell infiltration is evident, whereas vaccinated mice retained their alveolar structure with a modest presence of immune cells. Upon closer examination, a significant proportion of infiltrating immune cells in all groups were identified as lymphocytes, with smaller numbers of eosinophil and histocytes also present in the lungs (Figure S5).

We further performed detailed characterization of vaccination effects without viral challenge. In time-dependent profiling, spike-specific IgG levels remained high more than half a year after the boost, showing durable humoral immune responses after naked mRNA jet injection (Figure 2G). To directly demonstrate the neutralizing potential of PYRO-induced antibodies against SARS-CoV-2, mouse plasma obtained 2 weeks after the boost was subjected to a surrogate virus neutralization test (sVNT). sVNT evaluates the capability of the plasma to inhibit the interaction between the receptor-binding domain (RBD) of the SARS-CoV-2 spike protein and human angiotensin-converting enzyme 2 (hACE2) on the enzyme-linked immunosorbent assay (ELISA) plate.^38^ Figure 2H shows 50% inhibition titers in sVNT, revealing that PYRO-injected naked *spike* mRNA successfully inhibited the binding between spike RBD proteins and ACE2, especially at the 30-μg dose. We also evaluated the cellular immunity induced by PYRO injection of naked s*pike* mRNA. PYRO generated IFN-γ-positive splenocytes reactive with spike protein epitopes in vaccinated mice in a dose-dependent manner, with the highest spot number observed at 30 μg (Figure 2I). While *spike* mRNA was injected into BALB/c mice in the experiments above, C57BL/6J similarly produced spike-specific IgG titers and cellular immunity in a dose-dependent manner after PYRO injection of naked *spike* mRNA (Figure S6). Notably, the induction of humoral and cellular immunity using two different mRNA sequences with different sizes (4,247 nt for *spike* mRNA vs. 1,437 nt for *OVA* mRNA) demonstrates the robustness of jet injection in internalizing a wide range of naked mRNA in the skin for effective vaccination.

In the vaccination experiments described above, mRNA was dissolved in a hypotonic 10-mM HEPES buffer for PYRO injection. Here, we investigated the impact of buffer types on vaccination efficiency using *spike* mRNA, dissolved either in 10 mM HEPES buffer or isotonic Ringer’s lactate solution. Both buffer groups exhibited comparable levels of anti-spike IgG, while the Ringer’s lactate group exhibited enhanced cellular immune responses compared with the 10-mM HEPES group (Figures S7A and S7B). In an *fLuc* mRNA reporter assay, naked mRNA dissolved in Ringer’s lactate solution tended to exhibit a certain but nonsignificant enhancement of fLuc expression efficiency in the mouse skin compared with that dissolved in HEPES buffer (Figure S7C). A previous study also revealed the benefits of using Ringer’s lactate as an injection solution for naked mRNA in increasing the protein expression efficiency after i.d. injection.^39^ This potential enhancement of antigen expression efficiency by using Ringer’s lactate could enhance the induction of cellular immunity; however, a comprehensive understanding of the buffer effect on vaccination requires further investigation. This finding led us to use Ringer’s lactate as a buffer in the following NHP studies.

### PYRO-injected naked mRNA produces robust humoral immunity in NHPs

**Figure 3:**
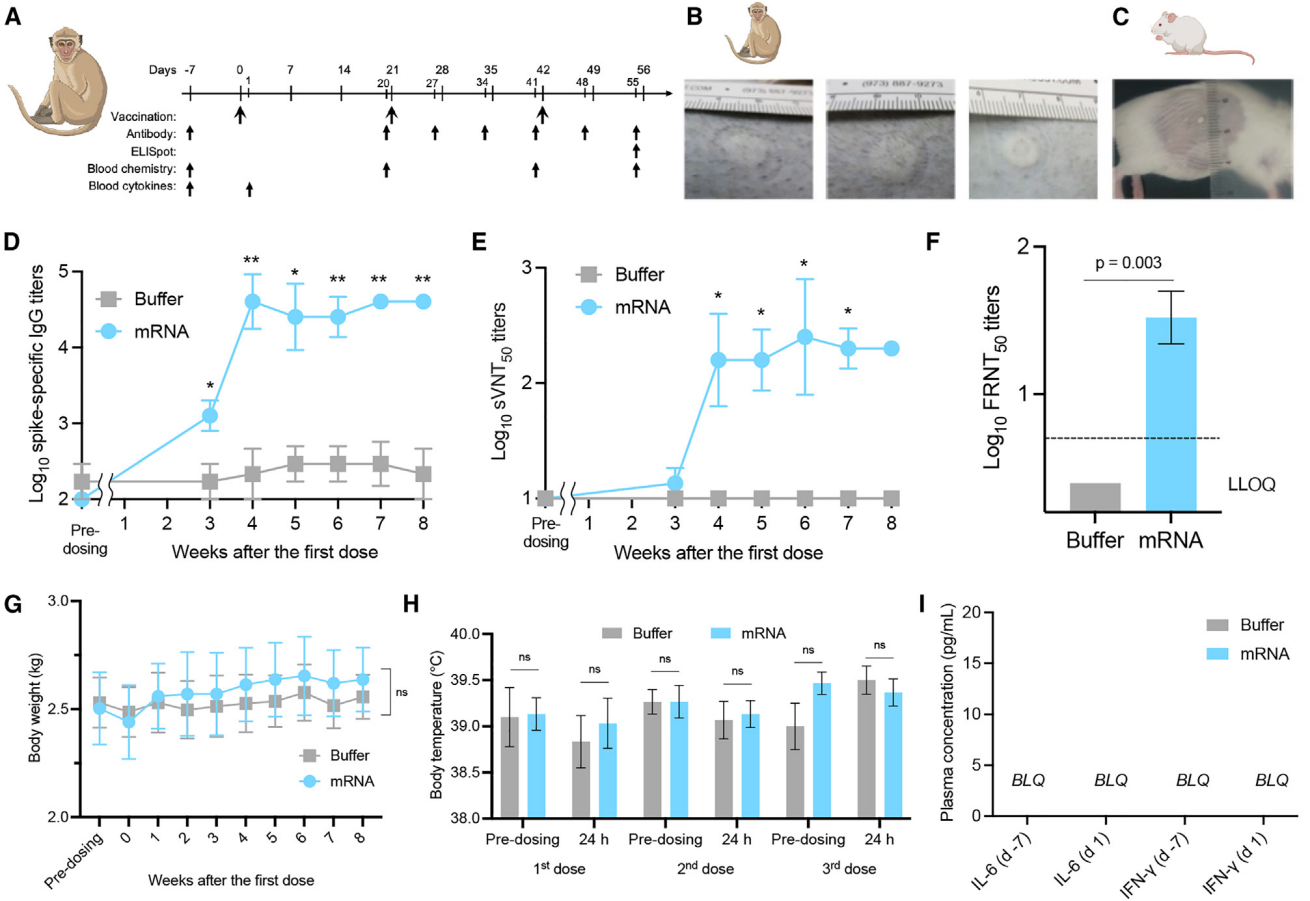
PYRO-injected naked *spike* mRNA vaccine in NHPs (A) Immunization and sample collection schedule. (B) The appearance of PYRO injection site on the back of Cynomolgus monkeys and (C) mice. (D) Log-transformed spike-specific IgG titers in NHPs PYRO-injected with buffer or mRNA solution. (E) Log-transformed sVNT measurement of plasma titer required for 50% inhibition of binding between ACE2 and SARS-CoV-2 RBD. (F) Log-transformed plasma titer required for 50% inhibition of SARS-CoV2 infection to VeroE6/TMPRSS2 cells (FRNT_50_ titer) 8 weeks after the first dose. (G–I) Systemic reactogenicity in PYRO-injected NHPs receiving either buffer or *spike* mRNA. (G) Change in body weight. (H) Body temperature measured immediately before and 24 h post-dosing, after each of the three doses. (I) Plasma levels of proinflammatory cytokines before and 24 h after the first dose. LLOQ, lower limit of quantification; n.s., nonsignificant; BLQ, below the limit of quantification. Data represent the mean ± SEM (n = 3), unpaired Student’s t test.

Further, the utility of naked mRNA PYRO injection was tested in NHPs. Cynomolgus monkeys received a total of three PYRO injections of naked *spike* mRNA solution separated by 3-week intervals (Figure 3A). The injection volume was 50 μL per dose in monkeys and 20 μL in mice. Intriguingly, PYRO-injected mRNA solution distributed widely in NHPs, with approximately 1 cm in diameter (Figure 3B). In contrast, the injection in mice provided only a 2-mm-sized area of distribution (Figure 3C). The wide distribution of the mRNA solution in the dermal layer in NHPs could potentially facilitate the contact of mRNA with APCs because the epidermis and dermis have high APC density. The skin bleb in NHPs exhibited a reduction in size within several hours post PYRO injection and became nearly invisible within 24 h in NHPs regardless of the presence of mRNA in the buffer. NHPs receiving three doses of 100 μg naked *spike* mRNA efficiently produced IgG antibodies against spike proteins (Figure 3D), with high neutralization activity to inhibit the binding of RBD and ACE2 in sVNT (Figure 3E). Note that sVNT titer measurement in mice and NHPs was performed using the same experimental condition, allowing us to compare the results obtained for these two species directly. Intriguingly, vaccinated NHP plasma exhibited strong neutralization potential comparable with those of vaccinated mice (Figures 2H and 3E). Further, NHP plasma capability to inhibit the viral infection to the cells was tested *in vitro* using an early circulating strain of SARS-CoV-2 viruses and VeroE6 constitutively expressing transmembrane protease, serine 2 (TMPRSS2) for facilitating the infection (Figure 3F). All three NHPs elicited efficient viral neutralization at plasma dilution of higher than 10-fold. Vaccinated NHPs showed a tendency of enhanced cellular immunity in IFN-γ ELISpot compared with the non-vaccinated control. However, the difference lacks statistical significance due to the high background values in the untreated control (Figure S8). As in mice, IL-4 spots were undetected (Figure S8).

NHPs were also monitored for signs of reactogenicity and toxicity throughout the 8 weeks of the study. PYRO-injected NHPs with buffer or mRNA solution showed no change in body weight (Figure 3G). The body temperature 24 h after the vaccination was comparable with that immediately before vaccination in the prime or the two boost doses (Figure 3H). The systemic release of proinflammatory cytokines such as IL-6 and IFN-γ 24 h after the prime was undetected in the blood (Figure 3I). Hematology and blood chemistry were comparable between mRNA-injected and buffer-injected groups (Tables S2 and S3). These data demonstrate high safety and robust antibody response following naked mRNA PYRO injection in NHPs. In the NHP experiment, we focused on comparing buffer-treated and PYRO injection groups without including the N&S group, as preceding mouse experiments demonstrated remarkable advantages of PYRO injection over N&S injection in vaccination (Figure 1). However, a future study should include the N&S group as a control to validate the benefit of PYRO injection in NHP vaccination.

### Antigen, but not mRNA, migrates to the LNs

**Figure 4:**
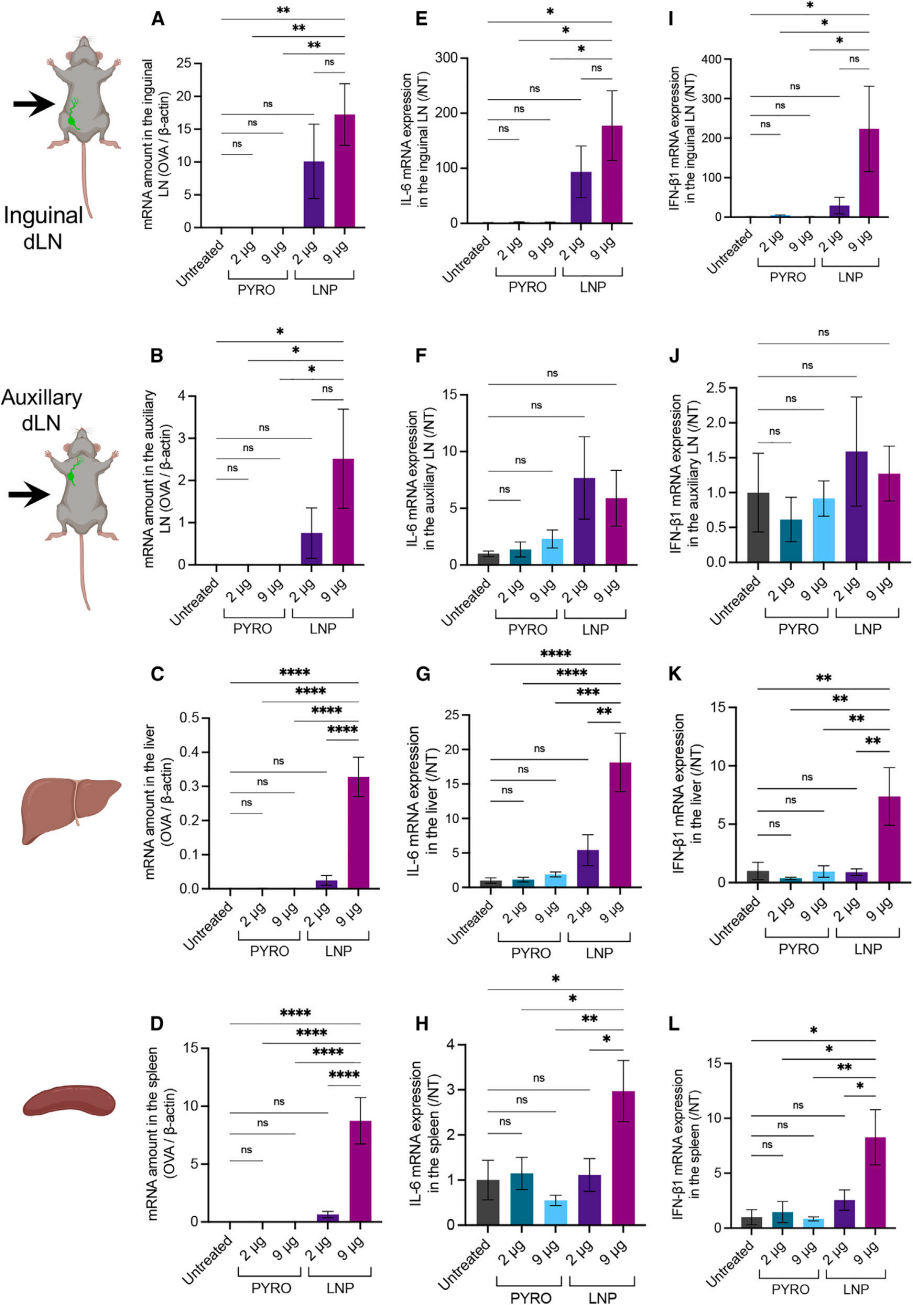
Biodistribution and safety analyses after injection of *OVA* mRNA Quantitative PCR (qPCR) was conducted 4 h after PYRO injection of naked mRNA or intradermal N&S injection of LNP. (A–D) *OVA* mRNA levels. Data are normalized to *β-actin* mRNA levels. (E–L) Transcript levels of *IL-6* (E–H) and *IFN-β* (I–L). Data are normalized to untreated samples. (A), (E), and (I) ipsilateral inguinal LN; (B), (F), and (J) ipsilateral auxiliary LN; (C), (G), and (K) liver; and (D), (H), and (L) spleen. Data represent the mean ± SEM (n = 5–6). Statistical analyses were performed by one-way ANOVA followed by Tukey’s post hoc test.

The following sections address comprehensive mechanistic and safety studies of mRNA vaccines using mice. First, we analyzed the biodistribution of *OVA* mRNA using qPCR 4 h after PYRO injection of naked mRNA and N&S injection of mRNA-LNPs at mRNA doses of 2 and 9 μg. In both the inguinal and auxiliary LNs ipsilateral to the injection site, *OVA* mRNA was undetectable following PYRO injection of naked *OVA* mRNA into the skin at both doses (Figures 4A and 4B). On the contrary, LNP induced efficient accumulation of *OVA* mRNA in both inguinal and auxiliary LNs even at a dose of 2 μg. Further, we conducted the same measurement at earlier time points, specifically 30 min post-injection, as naked mRNA may undergo rapid degradation in the extracellular and intracellular milieu within 4 h after PYRO injection.^40^ Similar to observations at 4 h post-injection, both inguinal and auxiliary LNs exhibited an undetectable signal of *OVA* mRNA after PYRO injection of naked mRNA but a considerable signal after LNP injection (Figure S9). Subsequently, we evaluated *OVA* mRNA distribution to other vital organs, the liver and spleen by qPCR 4 h post-injection. PYRO-injected naked mRNA was undetectable in these organs, while LNP injection resulted in substantial mRNA accumulation in these organs (Figures 4C and 4D). Collectively, naked mRNA does not leak from its injection site after PYRO injection, in contrast to LNPs, which migrate to the LNs, liver, and spleen. The localized biodistribution of PYRO-injected mRNA may offer a safety benefit.

In the safety analyses, we investigated the proinflammatory responses in the liver, spleen, and inguinal and auxiliary LNs by measuring the transcript levels of *interleukin (IL)-6* and *interferon (IFN)-β* using qPCR. Four hours after PYRO injection of naked mRNA at doses of 2 and 9 μg, the transcript levels in these organs were comparable to those in the untreated group (Figures 4E–4L). However, the injection of mRNA-LNP elevated the levels of proinflammatory transcripts in all tested organs at the 9-μg mRNA dose. This result is consistent with previous studies reporting systemic adverse events after local injection of mRNA-LNP vaccines in the 5- to 10-μg dose range.^41^^,^^42^ Thus, the mRNA dose of 9 μg in LNP might be considered excessively high for safe vaccination, despite its substantial potential for antibody production (Figures 1D, 1E, and 1H). In contrast, the 9-μg dose in PYRO injection was tolerable without systemic inflammation. Therefore, the maximum tolerable doses may differ between LNP and naked mRNA. In this context, it is worth noting that both 2 μg mRNA-LNP injection and PYRO injection of 9 μg naked mRNA provided comparable humoral immune responses (Figures 1D, 1E, and 1H) with minimal systemic inflammation.

**Figure 5:**
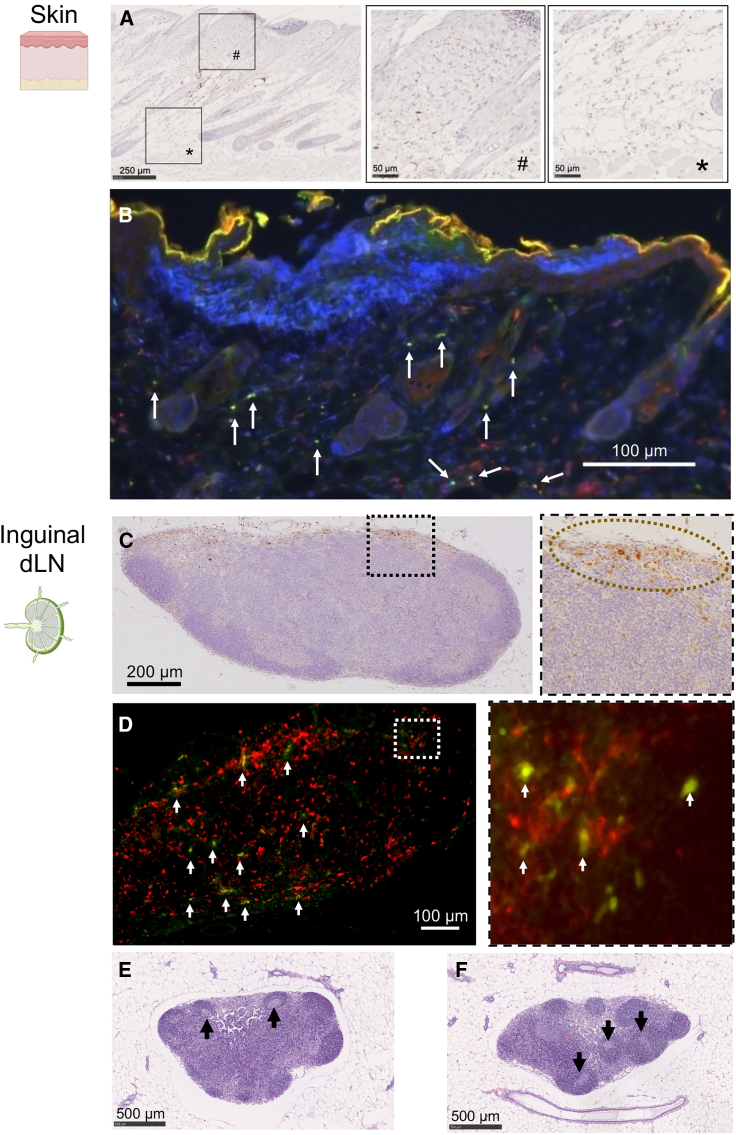
Antigen migration from skin to LNs (A–D) Distribution of EGFP expression in skin (A and B) and ipsilateral inguinal LNs (C and D) 24 h after PYRO injection of naked *EGFP* mRNA into mice. (A and C) EGFP was stained in brown by immunohistochemistry. (A) The middle and right images, magnifications of the left image, show the dermal and subcutaneous areas, respectively. (C) The right figures show the magnification of the dotted rectangle in the left figures. Dotted circles in the right figures highlight the area rich in EGFP-positive cells. (B and D) EGFP expression in dendritic cells was evaluated by staining EGFP in green and CD11c in red. White arrows indicate the colocalization of EGFP and CD11c. (E) and (F) H&E staining of the ipsilateral inguinal LN. Arrows indicate germinal centers. (E) 2 weeks. (F) 5 weeks.

The observed difference in biodistribution between naked mRNA and mRNA-LNPs raises questions about the mechanism by which naked mRNA PYRO injection elicits robust immune responses. Locally injected vaccines need antigen trafficking to the draining LNs to generate adaptive immunity. According to previous studies, current LNP-based mRNA vaccines have two main modes of action in antigen presentation at the draining LNs.^1^ (1) Resident immune cells, including APCs, take up LNPs at the injection site and migrate to the draining LNs.^43^ (2) LNPs distribute from the injection site to the draining LNs to introduce the antigen-encoding mRNA at the LNs.^11^ In PYRO injection of naked mRNA, the contribution of the latter mode is minimal, as naked mRNA migration to the LNs was undetected (Figures 4A and 4B). To investigate the former mode, we tracked the distribution of antigens from the injection site (skin in the flank) to the ipsilateral inguinal LN, a draining LN, by injecting *EGFP* mRNA as a reporter. PYRO-injected naked *EGFP* mRNA induced strong EGFP expression in the dermal and subcutaneous tissues at the injection site 24 h post-injection (Figure 5A). The distribution of EGFP expression is consistent with that of the injected solution visualized with black ink (Figure S3A). The EGFP signal co-localized in part with CD11c, a dendritic cell (DC) marker, indicating mRNA expression in APCs (Figure 5B). Meanwhile, the remainder of the EGFP signal did not co-localize with the CD11c signal, indicating the mRNA uptake and protein expression in other cell types. In the inguinal LN, the EGFP signal was also detected 24 h post-injection (Figure 5C). Notably, CD11c+ DCs expressed EGFP in the LN (Figure 5D), even in the absence of mRNA accumulation to the LN (Figures 4A and 4B). These results suggest that DCs taking up antigen mRNA in the skin migrate to the draining LN, wherein they interact with T cells for antigen presentation. We also observed structural maturation of inguinal LN after vaccination using PYRO-injected naked *OVA* mRNA. H&E staining of the LN sections revealed the formation of germinal centers 2 weeks after the prime (Figure 5E) and 2 weeks after the boost (Figure 5F). The LN maturation may enable long-lived antibody response following jet injection of naked mRNA vaccine (Figure 2G).^44^

Previous studies of i.d. LNP injection could provide valuable insights to these observations following naked mRNA PYRO injection (Figure 5). Similar to PYRO-injected mRNA, i.d.- injected LNPs provided protein expression to DCs at the injection site in the skin and draining LNs^43^ and induced the germinal center formation in the draining LNs.^45^ Notably, some of the DCs expressing antigen protein in the draining LNs displayed surface markers of dermal residual DCs, suggesting the migration of these DCs from the injection site to draining LNs after taking up antigen mRNA.^43^ Another study highlighted the role of dermal residual DCs, namely Langerhans cells and type 1 conventional DCs in intradermal LNP vaccines, showing that depletion of these DCs drastically impaired the vaccination effects.^46^ These findings support the important role of dermal residual DCs in mRNA vaccines, including PYRO injection of mRNA and i.d. LNP injection.

### PYRO injections work as a physical adjuvant

**Figure 6:**
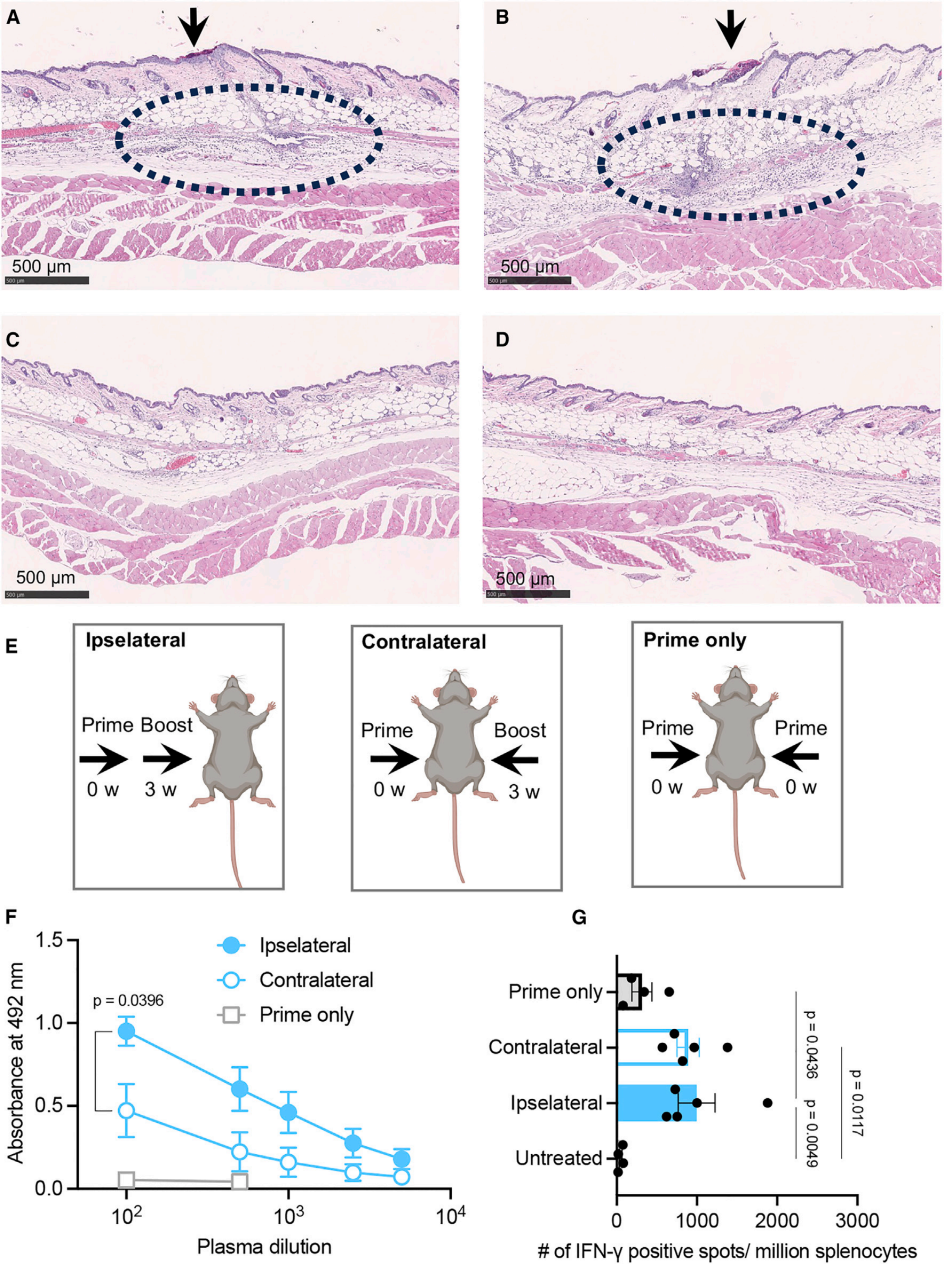
Physical adjuvant effect of PYRO (A–D) H&E staining of the skin at the injection site 24 h post-injection of mRNA solution by PYRO (A), buffer by PYRO (B), mRNA solution by N&S (C), and buffer by N&S (D). Arrows represent the injection site, and the dashed circles show the infiltrations of lymphocytes. (E) Experimental design to study the effect of injection location on vaccine effect. (F) OVA-specific IgG ELISA absorbance vs. dilution curves. Data represent the mean ± SEM (n = 5–6), unpaired Student’s t test. (G) Cellular immunity as measured by ELISpot assay using splenocytes of vaccinated mice. Data represent the mean ± SEM (n = 5–6), one-way ANOVA followed by Tukey’s post hoc test.

Inducing high antigen expression is not sufficient to induce a robust immune response. Indeed, an mRNA delivery system lacking adjuvant functionality fails to induce a strong immune response even with efficient antigen expression.^47^ In the present study, naked mRNA PYRO injection exhibited a robust vaccination effect without immunostimulatory adjuvants or immunostimulatory delivery materials, such as lipid components of LNPs. This motivated us to study the immunostimulatory mechanisms after the PYRO injection of naked mRNA. We observed the immunostimulatory events at the injection site after H&E staining of the skin sections. According to a previous study, immune cell infiltration into the skin peaks at 24 h after vaccination using adjuvants, leading us to choose 24-h post-injection as the observation time point.^48^ PYRO injection of naked mRNA induced localized lymphocyte infiltration at the injection site 24 h post-injection, demonstrating its adjuvant function (Figure 6A). Interestingly, similar proinflammatory reactions were observed after PYRO injection of the buffer without mRNA (Figure 6B). These observations indicate that mRNA immunostimulatory property minimally contributes to the adjuvant effects of naked mRNA PYRO injection. Indeed, the mRNA used in this study is N1-methyl-pseudouridine-modifed and thus low immunostimulatory. In contrast to PYRO injection, N&S injection of mRNA solution or buffer failed to provide lymphocyte infiltration (Figures 6C and 6D). These results indicate that PYRO injection, rather than mRNA properties, triggers proinflammatory reactions, which can function as a physical immunostimulatory adjuvant. From a safety viewpoint, it is noteworthy that these proinflammatory reactions and tissue damage were localized to a few-millimeter area, as indicated by dotted circles in Figures 6A and 6B and magnified images (Figure S10). In addition, these histological changes were mitigated by day 3 and completely resolved by day 7 (Figure S11).

Considering the contribution of the local immune reactions shown in Figure 6A, it became reasonable to assume that the location of PYRO injections might influence the vaccination outcomes. To clarify this point, we studied the position effect of the prime and boost doses using the following two mouse groups: one group received the boost on the same side as the prime (the ipsilateral group), while the other group received the two doses on opposite sides (the contralateral group) (Figure 6E). The ipsilateral boost group induced higher levels of OVA-specific IgG compared with the contralateral boost group (Figure 6F). However, the effect on cellular immunity was not as evident as that on humoral immunity following PYRO injection of naked mRNA, showing comparable levels of cellular immunity between ipsilateral and contralateral boosting (Figure 6G).

Two plausible mechanisms could explain the benefit of ipsilateral boosting in inducing humoral immunity. First, alternation in the skin microenvironment, such as immune cell infiltration, may contribute to the higher efficacy of the ipsilateral boost dose. Second, triggering immune responses in the same draining LN through prime and boost doses may play a critical role. Regarding the former possibility, immune cells intensively infiltrated the injection site on day 1 (Figure 6A), but these inflammatory changes were mitigated by day 3 and entirely diminished by day 7 (Figure S11). Contrary to the first hypothesis, this observation indicates that the skin microenvironment is normalized at the time of boost injection. Therefore, the benefit of ipsilateral vaccination is likely attributable to the second mechanism, involving draining LNs. Interestingly, a previous human study revealed the benefit of ipsilateral boosting following i.m. injection of mRNA-LNPs,^49^ aligning with our observation following PYRO injection of naked mRNA.

We also injected mice with 18 μg naked *OVA* mRNA at a single time point (prime only) to test the effect of repetitive administration. The prime-only administration schedule failed to induce anti-OVA IgG and cellular immunity as efficiently as observed in injecting mice with 9 μg per prime and boost (Figures 6F and 6G). A previous LNP study also showed the advantage of a prime-boost protocol over a prime-only protocol in vaccination using BNT162b2 COVID-19 mRNA.^50^

## Discussion

The skin is a highly immunogenic site for vaccination. Herein, we used a liquid jet injector (LJI) to facilitate mRNA delivery to the skin cells. Conventional LJI actuators, including those of the Lorenz force, are bulky and incapable of precisely controlling the injection depth.^51^^,^^52^ In contrast, PYRO is a portable LJI operated by a miniaturized single actuator. More importantly, PYRO precisely controls the injection pressure using a bi-phasic explosion. Both explosions generate gas, exerting pressure on the plunger of the liquid container to inject solutions from the nozzle tip. According to a previous force-profiling study, an initial explosion creates a hole in the skin across the stratum corneum with the penetration depth controlled by the amount of explosive.^28^^,^^29^ In the absence of a second explosion, the rise in liquid pressure persisted for approximately 10 ms. In contrast, with a second explosion, the duration of liquid pressure on the skin extended to approximately 100 ms. This prolonged pressure from the second explosion helped counteract the backflow of injected solution in the skin, facilitating wide dispersion of the solution from the hole created by the initial explosion.

The amount of gunpowder for each explosion was optimized for different animal species, considering variations in skin thickness, elasticity, and viscoelasticity.^53^ This optimization enabled controlled distribution of the solution in the dermal and subcutaneous spaces across various species, including mice, rats, and pigs.^54^^,^^55^ In the present study, we used two types of explosive cartridges, each optimized for jet injection in either mice or NHPs. In mice, the solution predominantly dispersed within the dermal and subcutaneous tissues (Figure S3A) and induced protein expression there (Figure 5A). Future studies should investigate the distribution of mRNA solution in NHP skin and the influence of the drug types on the distribution profile to enhance our understanding of PYRO injection.

PYRO might offer additional benefits, potentially addressing several issues associated with N&S i.d. vaccination, such as the difficulty in administering hypodermic needle injections and needle phobia.^24^^,^^56^ Indeed, a previous phase I clinical study of naked plasmid DNA PYRO injection reported no injection failure, successfully delivering up to 400 μL of solution in humans.^57^ This volume exceeds that used in the present study (20 μL in mice and 50 μL in LNPs), enabling larger mRNA doses in future clinical trials. Furthermore, the severity of pain or other adverse events at the injection site were modest, revealing a high level of tolerability for the PYRO injection procedure in humans.

In the present study, physical forces of the PYRO injection improved the vaccination effect of naked mRNA by the following two plausible mechanisms: (1) enhancing the delivery efficiency of naked mRNA (Figures 1A and 1B) and (2) inducing proinflammatory responses at the injection site, which may serve as an immunostimulatory adjuvant (Figures 6A–6D). Notably, liquid flow rate provided by PYRO (1 mL/s) is more than 40-fold faster than that provided by N&S injection (0.025 mL/s at maximum), applying drastically enhanced shear stress to the skin tissue.^28^^,^^29^ The shear stress may facilitate the cellular uptake of mRNA for the former mechanism and activate danger signals in the skin to provoke proinflammatory responses for the latter. Regarding the latter, PYRO elicited lymphocyte infiltration into the injection site even without mRNA, whereas N&S injection of mRNA or buffer failed to induce such responses (Figures 6A–6D). This observation indicates that physical stress, rather than mRNA immunostimulatory properties, triggers immune responses. Previous studies have also addressed related phenomena. For example, several studies reported the proinflammatory role of the blood-flow-derived shear stress on endothelial cells,^58^^,^^59^ although the strength and duration of the shear stress are largely different between blood flow and PYRO injection. PYRO instantaneously produces strong shear stress. In addition, previous vaccine studies revealed the potential roles of physical stress in inducing localized immune infiltration, which acts as a vaccine physical adjuvant. The triggers of physical stress include electroporation,^60^ vacuum electroporation,^61^ microneedles,^62^^,^^63^ an autoinjector with a needle array,^64^ and laser light.^65^ These studies support our finding that PYRO injection plays an immunostimulatory role in vaccination. In the future study, we need to clarify the later immunization steps bridging the PYRO-driven innate immune responses to the adaptive immune responses and vaccination outcome.

From a safety viewpoint, controlling the distribution of mRNA and antigen is a critical issue of local injection. Previous studies tackled this issue with several strategies. For instance, one strategy adds microRNA target sequences to mRNA untranslated regions to prompt mRNA degradation in the off-target tissues,^66^ and another strategy develops delivery carriers with minimal systemic distribution.^14^^,^^15^ Our unique approach of using naked mRNA utilizes mRNA nuclease susceptibility to avoid the systemic distribution of mRNA, supported by the fact that naked mRNA is rapidly degraded after systemic distribution.^67^ Indeed, qPCR measurement did not detect intact mRNA in the draining LNs, liver, or spleen following PYRO injection of naked mRNA (Figures 4A–4D and S9). This strategy also avoids the potential safety risks of using delivery materials, such as immunostimulatory lipids in LNPs, which distribute systemically. As a result, naked mRNA did not induce proinflammatory responses in the draining LNs, liver, or spleen of vaccinated mice (Figures 4E–4L). These safety profiles of naked mRNA vaccines might solve several concerns of current mRNA vaccines, including rare cases of hepatic autoimmunity,^68^^,^^69^ myocarditis,^70^ hyperthyroidism,^71^ and allergic reactions.^72^ Meanwhile, systemic distribution of mRNA, especially in the lymphoid organs, e.g., the spleen and LNs, is considered to play a critical role in current LNP-based mRNA vaccines.^11^ However, naked mRNA PYRO injection provided robust immunization even without distribution to the lymphoid organs. In our mechanistic experiments using *EGFP* mRNA as a representative of antigen mRNA, EGFP-positive APCs existed in the draining LN after PYRO injection (Figure 5D), even without mRNA migration to the LN (Figures 4A, 4B, and S9). This result indicates that APCs that take up the antigen mRNA in the skin migrate to the draining LN, wherein APCs interact with T cells. This mechanism may contribute to the efficient induction of humoral and cellular immunity by jet injection.

In a SARS-CoV-2 challenge experiment, PYRO injection of naked *spike* mRNA reduced viral load and alleviated tissue damage in mouse lungs (Figure 2). This is a pioneering report demonstrating the protective effect of naked mRNA vaccines in an infectious disease model. Furthermore, PYRO-injected naked mRNA induced efficient humoral immunity even in NHPs with efficient ACE2 binding inhibition in sVNT comparable with that in mice (Figures 2H and 3E). The wide distribution of mRNA solution in the dermal layer, rich in APCs, may explain robust vaccination in NHPs (Figure 3B). Moreover, NHP plasma effectively prevented *in vitro* SARS-CoV-2 infection in cultured cells (Figure 3F). Such efficient neutralizing activity is favorable in COVID-19 vaccines, as neutralizing activity is strongly correlated with vaccine effectiveness in preventing infection of SARS-CoV-2 in the clinic.^73^^,^^74^ In terms of storage stability, naked mRNA maintains full *in vitro* protein expression activity when stored at temperature of 4°C in water or the lyophilized form for at least a month.^75^ Lyophilization enhances the storage stability of naked mRNA, preserving its integrity over 6 months,^76^ similar to the stability of lyophilized mRNA-LNPs.^77^ For clinical translation, we plan to study the protective effect of PYRO-injected naked mRNA against more relevant variants of SARS-CoV-2, comparing its safety and efficacy with LNPs. In addition, future studies should aim to boost the potency of our naked mRNA vaccine by optimizing mRNA chemical modifications, immunostimulatory adjuvants, and dosing schedule.

## Materials and methods

### Materials

CleanCap *EGFP* mRNA, N1-methyl-pseudouridine (m1Ψ)-modified *OVA* mRNA, and m1Ψ-modified *spike* mRNA with di-proline substitutions of K968 and V969 were obtained from Trilink Biotechnologies (San Diego, CA, USA). The same sequence utilized in BNT162b2, an approved mRNA vaccine, was used.^78^ The Actranza lab i.d. delivery device (PYRO) was purchased from Daicel Corporation (Tokyo, Japan). Ovalbumin (OVA) was purchased from Sigma-Aldrich (St. Louis, MO, USA). Goat anti-mouse IgG, IgG2a, and IgG1 horseradish peroxidase (HRP)-conjugated antibodies were bought from Abcam (Cambridge, UK), R&D systems (Minneapolis, MN), and Cytiva (Tokyo, Japan). Anti-mouse IgG HRP-conjugated antibody was obtained from Cytiva in Figure 2B, from R&D Systems in Figure 6F, and Abcam in the other experiments. Anti-IFNγ and anti-IL-4 ELISpot PLUS kits were purchased from Mabtech (Nacka Strand, Sweden). PepTivator Ovalbumin epitope mix was provided by Miltenyi Biotec (Nordrhein-Westfalen, Germany). PepMix SARS-CoV-2 (S) was obtained from JPT Peptide technologies (Berlin, Germany). Paraformaldehyde (PFA, 16%) was purchased from Alfa Aesar (Haverhill, MA, USA). SARS-CoV-2 Spike RBD-ACE2 Blocking Antibody Detection ELISA Kit was obtained from Cell Signaling Technologies.

### mRNA synthesis

*Luciferase* DNA was constructed by cloning luciferase gene (*luc2*) (pGL4.13, Promega, Madison, WI), 120 adenine, and BsmBI cut sites into pSP73 plasmid vector. The plasmid was amplified in *E. coli* DH5α competent cells (Takara Bio Inc., Otsu, Japan), and then extracted and purified using a Nucleobond xtra maxi plus EF (Takara bio). Plasmids were linearized and fragmented by incubation with BsmBI overnight at 55°C, and the desired DNA fragment was separated by gel electrophoresis and extracted using a gel extraction kit (Qiagen, Hilden, Germany). The extracted DNA was further treated with T4 DNA polymerase (Takara Bio Inc., Otsu, Japan) for DNA blunting. Finally, *in vitro* transcription (IVT) synthesis of mRNA was carried out using MEGAscript T7 Transcription Kit (Waltham, MA, USA) with the addition of ACRA 5′ cap (Trilink Biotechnologies) and m1Ψ-5′-triphosphate (Trilink Biotechnologies) instead of uridine bases. The reaction was allowed to proceed for 1 h at 37°C, and the transcribed mRNA was purified using RNeasy Mini Kit (Qiagen, Hilden, Germany). The quality of mRNA was checked using a Bioanalyzer (Agilent Technology, CA, USA). Note that *OVA*, *Spike*, and *EGFP* mRNA, prepared using the CleanCap method, were purchased from Trilink Biotechnologies.

### LNP formulation

In a four-component ionizable lipid nanoparticle (LNP) preparation, an ethanolic solution of D-Lin-MC3-DMA, DSPC, cholesterol, and PEG2000-DMG (50:10:38.5:1.5 mol %) was rapidly mixed with 3 volumes of 50 mM sodium citrate buffer (pH = 3) containing the mRNA at [amino groups in D-Lin-MC3-DMA (N)]/[phosphate groups in mRNA (P)] ratio of 5, using a microfluidic micromixer (NanoAssemblr, Precision NanoSystems Inc., Vancouver, BC, Canada) at a flow rate of 12 mL/min. The product was then diluted 40x in PBS and concentrated using 30-kDa centrifugal filters (Millipore, MA, USA) to remove the ethanol. Encapsulation efficiency was determined using Quant-it RiboGreen RNA Assay Kit (Thermo Fisher Scientific, Waltham, MA, USA). Particle size was measured with dynamic light scattering using a diode laser (λ = 532 nm) with a scattering angle of 173° (Zetasizer Nano-ZS, Malvern Instruments, Worcestershire, UK). The LNP had the size of 67 nm, almost neutral ζ-potential, and more than 90% encapsulation efficiency (Table S4).

### PYRO injection

Mouse experiments were performed under the ethical guidelines of the Innovation Center of NanoMedicine (iCONM), Kawasaki Institute of Industrial Promotion (Kanagawa, Japan). NHP experiments were performed by Ina Research Inc. (Nagano, Japan) under Act on Welfare and Management of Animals and animal experimental guidelines (Nagano, Japan) after being approved by the Institutional Animal Care and Use Committee in Ina Research Inc. For PYRO injections, mouse fur was shaved. In the case of mice, Actranza lab device,^28^^,^^29^^,^^54^^,^^55^ loaded with a 20-μL solution, was applied to the skin, ensuring that half of the tapered part is embedded in the skin (Figure S12). For NHPs, the solution volume increased to 50 μL, and the plastic cylinder surrounding the nozzle tip indicated the depth of the tip embedment. The solution volume for mice and NHPs was determined according to the manufacturer’s protocol. The optimization of jet power is tailored for this specific application force. The average jet power for NHPs was 2-fold higher than that for mice. The leakage of the injected solution from the skin is less than 1 μL out of 20 μL injection volume in mice and undetected in NHPs. This result is consistent with a previous porcine report, wherein the injection at optimal pressure resulted in almost no leakage.^55^ Note that we did not employ any special procedure to prevent mRNA degradation in the skin, such as the addition of an RNase inhibitor.

### IVIS imaging

Female C57BL/6J were obtained from Charles River Laboratories Inc., Yokohama, Japan, and used at 6–9 weeks of age. A luciferase reporter assay was used to quantify protein expression following i.d. delivery of 1 μg *luc2* mRNA. For N&S injections, mouse fur was removed using Epilat cream at one flank, and 20 μL HEPES buffer (10 mM, pH 7.4) containing naked *luc2* mRNA using a 35-gauge needle (FastGene Nano Needle, Nippon Genetics, Tokyo, Japan). Protein expression was then quantified at 4, 24, 48, and 72 h post-injection using an *in vivo* imaging system (IVIS, PerkinElmer). At each time point, imaging was performed 10 min after i.p. injection with 200 μL of 15 mg/mL luciferin substrate (Promega). The total flux of luminescence was calculated by gating a region of interest at the injection site, followed by subtraction of the background signal in each image.

### Mouse immunization studies

*OVA* mRNA or *spike* mRNA was dissolved in HEPES buffer (10 mM, pH = 7.3) and injected in the naked form. Twenty microliters of the solution containing a specific amount of mRNA was injected followed by a booster 3 weeks later. Two weeks after the boost, female BALB/c or C57BL/6J mice (Charles River Laboratories Inc.) were euthanized, and blood was collected from the inferior vena cava in heparinized tubes unless specified otherwise. Blood plasma was obtained by centrifugation at 2,000 × *g* for 10 min at 4°C and stored at −80°C until used as described in the section Detection of mouse antibodies. Spleens were also collected and processed for ELISpot assay as described in the section ELISpot assay in mice. The “untreated” group in these experiments did not receive buffer injection or any other treatment.

### Detection of mouse antibodies

Antibodies in the blood plasma were evaluated using enzyme-linked immunosorbent (ELISA). For the detection of antigen-specific antibodies, OVA or recombinant spike protein was dissolved at 2 μg/mL in carbonate buffer (50 mM, pH = 9.6). Fifty microliters per well of protein solution was added into Clear Flat-Bottom Immuno Nonsterile 96-Well Plates (Thermo). After overnight incubation at 4°C, the plates were washed three times with 0.5% w/v Tween 20 in PBS (PBS-T). A 100 μL diluent of 1% BSA and 2.5 mM EDTA in PBS-T was added to each well and further incubated for 1 h at 23°C. After removing the diluent, 50 μL blood plasma that was serially diluted in the same diluent was added to each well. The plates were incubated overnight at 4°C, before washing three times with PBS-T. Fifty microliters of goat anti-mouse IgG (1:8,000), IgG1 (1:10,000), or IgG2a (1:10,000) HRP-conjugated antibodies was added to each well and incubated for an additional 2 h at 23°C. Finally, 100 μL/well of the HRP substrate was added and incubated for 30 min at 23°C away from light. The reaction was stopped by adding 2M sulfuric acid and measuring the absorbance of 492 nm using a plate reader (Tecan, Switzerland). Titers were determined as the highest dilution that showed absorbance optical density >0.1.

The detection of antibodies that block the interaction between the RBD of the SARS-CoV-2 spike protein and angiotensin-converting enzyme 2 (ACE2) was measured by sVNT using a SARS-CoV-2 Spike RBD-ACE2 Blocking Antibody Detection ELISA Kit following the manufacturer’s protocol. Titers were determined as the highest dilution that showed binding inhibition >50%.

### ELISpot assay in mice

Spleens from immunized mice were collected and disintegrated separately using a steel grid mesh with 5 mL of RPMI-1640 medium containing 10% fetal bovine serum, 1 mM sodium pyruvate, 10 mM HEPES, 50 μM mercaptoethanol, and 1% penicillin/streptomycin. The suspension was then passed through a 40-μm nylon mesh (Cell strainer, Falcon) to form a single-cell suspension. Splenocytes were seeded at a density of 2.5 × 10^5^ cells/well in anti-IFNγ or anti-IL-4-ELISpot 96 well plates and stimulated by the addition of 10 μL epitope mixture dissolved in PBS (0.025 μg/well OVA epitope mix and 0.2 μg/well spike epitope mix). The plates were incubated at 37°C and 5% CO_2_ overnight. The next day, the plates were washed and treated according to the manufacturer’s protocol. Spots were counted on an ELISpot plate reader (AID GmBH, Germany).

Manual MACS Magnetic Separator (Miltenyi Biotec) was used to separate CD4+ and CD8+ T lymphocytes from the cell suspension following the manufacturer’s instructions. CD4+ or CD8+ splenocytes were seeded at a density of 2.5 × 10^5^ cell/well in anti-IFNγ ELISpot 96-well plates. To detect the number of OVA-reactive CD4+ or CD8+ cells, OVA epitopes were presented on the surface primary DC obtained from donor mice. Primary DCs were harvested from mice femur and cultured according to a previous protocol.^79^ Primary DCs were cultured in 12-well plates at a density of 2.5 × 10^5^ cells/well and incubated with 1 μg OVA epitope mix overnight. Fifty microliters containing 15,625 primary DCs pulsed with OVA epitope were then added to CD4+ or CD8+ cells splenocytes and further incubated overnight. Plates were further treated, and spots were counted as described above.

### Viral challenge experiment

The SARS-CoV-2 TY/WK-521/2020 strain (GISAID ID: EPI_ISL_408667) was provided by Dr. Masayuki Saijo, Mutsuyo Takayama-Ito and Masaaki Satoh (Department of Virology 1, National Institute of Infectious Diseases), and was subcultured in Vero E6/TMPRSS2 cells (JCRB1819, Japanese Collection of Research Bioresources [JCRB] Cell Bank, National Institute of Biomedical Innovation, Health and Nutrition, Osaka, Japan) and grown in DMEM (Nissui Pharmaceutical Co. Ltd., Tokyo, Japan) supplemented with 10% inactivated fetal bovine serum, penicillin (100 units/mL), streptomycin (100 μg/mL), and G-418 (1 mg/mL). All procedures using SARS-CoV-2 were performed in biosafety level 3 facilities by personnel wearing powered air-purifying respirators (Shigematsu Co., Ltd., Tokyo, Japan).

Female BALB/c mice (Japan SLC Inc., Shizuoka, Japan) were vaccinated twice every 3 weeks. Two weeks after the boost, mice were infected with 1 × 10^5^ PFU/50 μL/animal of SARS-CoV-2 early circulating strain (TY/WK-521). Five days before virus infection, mice were inoculated intranasally with 5 × 10^7^ FFU/animal of rAd5 hACE2.^34^^,^^80^ The body weight of each mouse was monitored, and the loss of 30% of initial body weight was defined as the endpoint for euthanasia. All animals were euthanized at 5 days post infection, and blood and lungs were then collected.

The lungs of mice were homogenized using a Multi-Beads Shocker (Yasui Kikai, Osaka, Japan) in nine volumes of Hanks’ Balanced Salt Solution (Gibco #14025-092). Total RNA was extracted from lung homogenates using an RNeasy Mini Kit (Qiagen) according to the manufacturer’s instructions. The levels of RNA corresponding to the N protein-encoding gene of SARS-CoV-2 were measured using the TaqMan Fast Virus 1-Step Master Mix (Thermo Scientific). Each 20-μL reaction mixture contained 5.0 μL of 4× TaqMan Fast Virus 1-Step Master Mix, 0.25 μL of 10 μM probe, 1.0 μL each of 10 μM forward and reverse primers, 7.75 μL of nuclease-free water, and 5.0 μL of nucleic acid extract. Amplification was carried out in 96-well plates using a CFX-96 cycler equipped with CFX Maestro software (Bio-Rad, Hercules, CA, USA). The thermocycling conditions were as follows: 5 min at 50°C for reverse transcription, 20 s at 95°C for the inactivation of reverse transcriptase and initial denaturation, and 45 cycles of 5 s at 95°C and 30 s at 60°C for amplification. Each run included a no-template control reaction as well as reactions intended to provide a standard curve. The latter used *in vitro* transcribed RNA of the N protein-encoding gene (at 10^0^, 10^1^, 10^2^, 10^3^, 10^4^, 10^6^, and 10^8^ copies/reaction); this template was generated from the cDNA of SARS-CoV-2 AI/I-004/2020 using the T7 RiboMAX Express Large Scale RNA Production System (Promega, Madison, WI, USA). The primers and probe used to detect the WK-521 strain were as follows: forward primer, 5′-GACCCCAAAATCAGCGAAAT-3′; reverse primer, 5′-TCTGGTTACTGCCAGTTGAATCTG-3′; and probe, 5′-(FAM)-ACCCCGCATTACGTTTGGTGGACC-(BHQ-1)-3′.

The infectious SARS-CoV-2 titer was determined using a standard plaque assay. Briefly, the left lung lobe from each mouse was weighed and homogenized in nine volumes of Hanks-balanced saline solution (Gibco #14025-092) using a Multi-Beads Shocker. The homogenates were centrifuged at 3,000 × *g* for 10 min at 4°C. The supernatant was collected and stored at −80°C until use. Serial 10-fold dilutions of the supernatant or virus solutions (100 μL per each well) were inoculated onto confluent monolayers of Vero E6/TMPRSS2 cells in six-well plates and incubated at 37°C for 1 h. Unbound viruses were removed by washing the cells with DMEM. The cells were then overlaid with DMEM containing 10% inactivated fetal bovine serum and 0.6% agarose (Sigma-Aldrich, St. Louis, MO, USA). After 48 h of incubation at 37°C, the cells were fixed in 10% neutral buffered formalin and stained with 1% crystal violet. The titer of SARS-CoV-2 was defined as plaque-forming units per gram of lung tissue (PFU/g lung) or PFU per milliliter (PFU/mL). The detection limit is 100 PFU/g lung for the lung homogenates or 10 PFU/mL for virus stock solutions.

In lung histopathology analyses, mouse lung samples were fixed in 10% neutral buffered formalin, embedded in paraffin, and cut into 4-μm-thick sections, which were mounted on glass slides. Tissue sections were stained with hematoxylin and eosin and observed by light microscopy.

### NHP immunization studies

Cynomolgus monkeys (female, 3–4 years old) received 50 μL of lactated Ringer’s buffer or mRNA solution (100 μg/monkey) at a single position on their back in each prime and boost dosing. ELISA assay was performed as described in section 5 except for using Goat Anti-Monkey IgG H&L (HRP) for detecting monkey IgG. Titers were determined as the highest dilution that showed absorbance optical density >0.15. sVNT was performed as described above in the section Detection of mouse antibodies. Neutralization assay of NHP plasma against SARS-CoV2 was performed by Bio Medical Laboratories, Inc. Briefly, the diluted plasma was incubated with an early strain of SARS-CoV-2 at the final viral concentration of 1 TCID_50_ (median tissue culture infectious dose)/μL for 1 h at 37°C. Then, the solution was added to VeroE6 cells constitutively expressing transmembrane protease, serine 2 (TMPRSS2) for 5-day incubation. Finally, the cell was stained with crystal violet for evaluating the cytopathic effect of SARS-CoV-2. Peripheral blood mononuclear cells (PBMCs) were collected for ELISpot assays using ELISpot Plus: Monkey IFN-γ (HRP) and ELISpot Plus: Human IL-4 (HRP) kits (Mabtech); 2 × 10^5^ PBMCs seeded onto 96-well plates in the kits were incubated with 1 μg of PepMixTM SARS-CoV-2 (Spike Glycoprotein) for 20 h followed by counting spot numbers using IMMUNOSPOT S6 Versa (Cellular Technology Ltd., Cleveland, OH). Safety analyses were performed using XN-2000 (Sysmex, Kobe, Japan), CA-510 (Sysmex), and flow cytometer (FACS Canto II, Becton, Dickinson and Company, Franklin Lakes, NJ) for measuring blood cell counts and coagulation functions, type 7180 auto analyzing machine (Hitachi, Tokyo, Japan) for blood chemistry analyses, and BDTM Cytometric Beads Array (CBA) Non-Human Primate Th1/Th2 Cytokine Kit (Becton Dickinson and Company) for blood cytokine measurement.

### Histopathology and microscopy

For the observation of protein expression in the skin following injection of 9 μg *EGFP* mRNA, around 1 cm^2^ of the skin surrounding the injection site was excised and fixed in 4% PFA in PBS for 24 h. The skin was then cut into several strips each around 2 mm in width, immersed in paraffin, and cooled down to form blocks for sectioning. Draining LNs proximal to the injection site were also collected 24 h post-injection and treated the same as described above. The samples were sliced at 4-μm thickness, stained with H&E, and observed under the optical microscope. EnVision system (DAKO) was applied for immunofluorescent staining of the paraffin-embedded samples with 5 min autoclaving at 121°C under pH 9.0. Overnight incubation was performed for labeling DCs by a rabbit anti-CD11c antibody (Cell Signaling, #97585, 500× dilution) and EGFP by goat anti-GFP antibody (Abcam, ab5450, 1,000× dilution). On the next day, the sections were washed, and a mixture of secondary antibodies composed of Alexa Fluor 568 rabbit anti-goat IgG and Alexa Fluor 488 goat anti-rabbit IgG (Invitrogen) were applied to labeled CD11c and EGFP, respectively. The sections were incubated for 1 h at room temperature, washed, and observed under the fluorescent microscope (Keyence, Osaka, Japan) after the addition of DAPI to stain the cell nuclei. For the observation of lymph node maturation and proinflammatory reactions in the skin, 10 μg of spike mRNA was injected and H&E staining was performed as described above.

### Quantitative PCR

For the quantification of *OVA* mRNA delivered to mouse organs following injection in the skin, LNs, liver, and spleen were extracted and homogenized by a Multi-Beads Shocker at 2,000 rpm for 30 s. Total RNA was extracted from the homogenates using an RNeasy Mini Kit (Qiagen, Hilden, Germany). After the removal of genomic DNA by enzymatic degradation, complementary DNA (cDNA) was obtained by reverse transcription using a ReverTraAce with a gDNA Remover Kit (TOYOBO, Osaka, Japan). The products were run on a 7500 fast real-time PCR (Applied Biosystems) using FastStart Universal SYBR Green Master Kit. For the quantification of *OVA* mRNA, a forward primer: GAACCAGATCACCAAGCCCA, and a reverse primer: GTACAGCTCCTTCACGCACT were used. Proinflammatory cytokines in these organs were also measured by quantitative PCR (qPCR), as described above, using the following primers. *IL-6* (Mm00446190_m1): 4331182, *IFN-β* (Mm00439552_s1): 4331182, and *Β-Actin*: 4352933E. Data were analyzed with a 2^−ΔCt^ method using the β-actin gene as an endogenous control housekeeping gene. The proinflammatory cytokines data were presented after normalization to untreated samples.

### Statistical analysis

The statistical significance between the two groups was analyzed using an unpaired, two-tailed Student’s t test. Multiple comparisons among three or more groups were performed using one-way ANOVA followed by Tukey’s post hoc test. Comparison against NT samples was performed using Dunette’s test. A statistically significant difference was set at p < 0.05.

## Data and code availability

All data are included in the paper or the supplemental information. Additional data are available from the corresponding authors on reasonable request.

## Supplemental information

Document S1. Figures S1–S12 and Tables S1–S4

Document S2. Article plus supplemental information
