# Supplementary material for: Carrier-free mRNA vaccine induces robust immunity against SARS-CoV-2 in mice and non-human primates without systemic reactogenicity
Source: Mol Ther. 2024 Apr 2;32(5):1266–83. doi: 10.1016/j.ymthe.2024.03.022 (PMC11081875; doi:10.1016/j.ymthe.2024.03.022)
Supplement: Document S1. Figures S1–S12 and Tables S1–S4 [file mmc1.pdf]

YMTHE, Volume *32*

Supplemental Information

Carrier-free mRNA vaccine induces robust immunity against SARS-CoV-2 in mice and non-human primates without systemic reactogenicity

Saed Abbasi, Miki Matsui-Masai, Fumihiko Yasui, Akimasa Hayashi, Theofilus A. Tockary, Yuki Mochida, Shiro Akinaga, Michinori Kohara, Kazunori Kataoka, and Satoshi Uchida

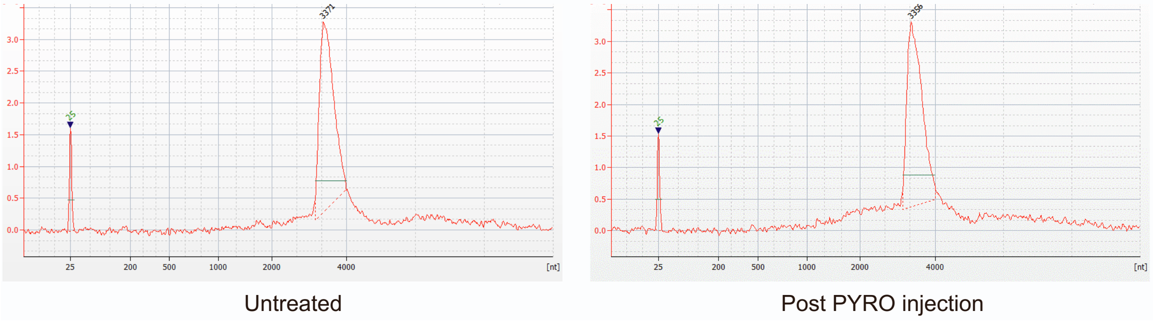

Figure S1. Integrity of *spike* mRNA following PYRO injection. PYRO-injected mRNA solution was collected in a plastic tube. The quality of mRNA samples with or without PYRO injection was assessed using Bioanalyzer (Agilent Technology, CA, USA), capillary electrophoresis. In this analysis, we selected *spike* mRNA (4,247 nt) due to its long length, enabling sensitive detection of mRNA degradation.

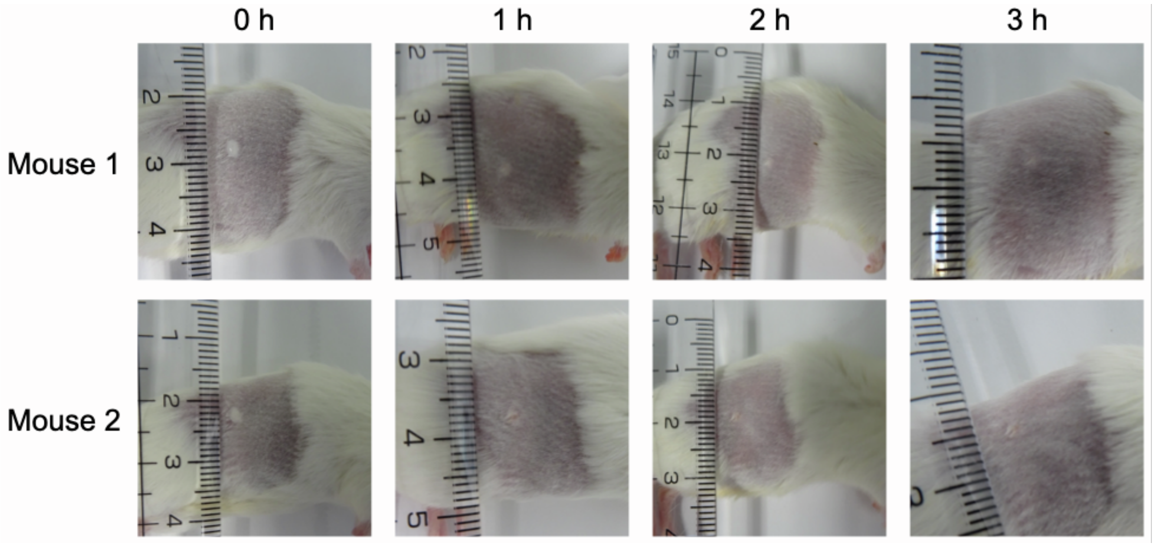

Figure S2. Time-dependent observation of mouse skin after PYRO-injection of mRNA solution.

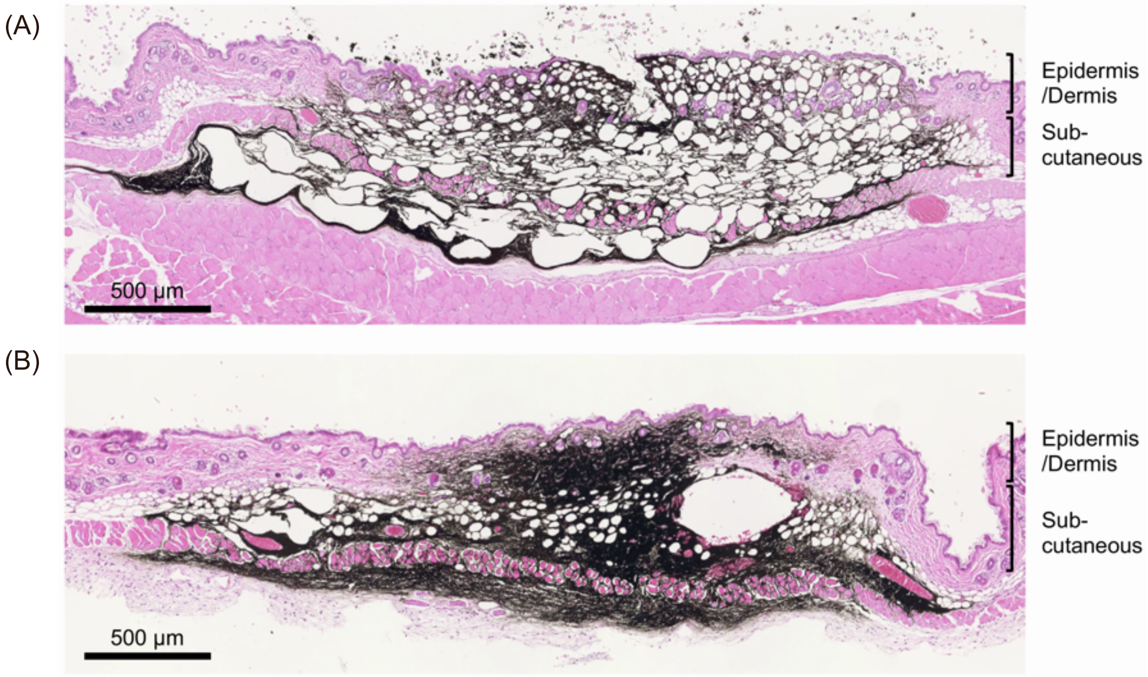

Figure S3. The solution distribution in the skin. Tissue sections were prepared immediately following i.d. injection of black ink. (A) PYRO injection. (B) N&S injection.

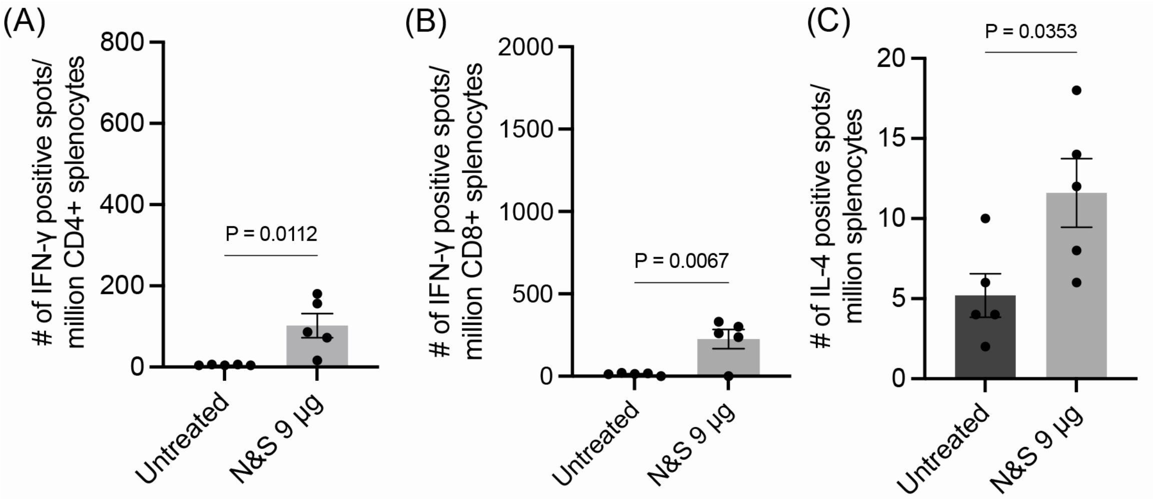

Figure S4. ELISpot assays following N&S injection with naked mRNA. *OVA* mRNA was injected twice at a 3-week interval at the dose of 9 μg in each injection, followed by ELISpot 2 weeks after the boost dose. (A and B) IFN-γ ELISpot for quantifying (A) CD4+ and (B) CD8+ T cell responses. (C) IL-4 ELISpot using whole splenocytes. The data represent the mean ± SEM (n = 5).

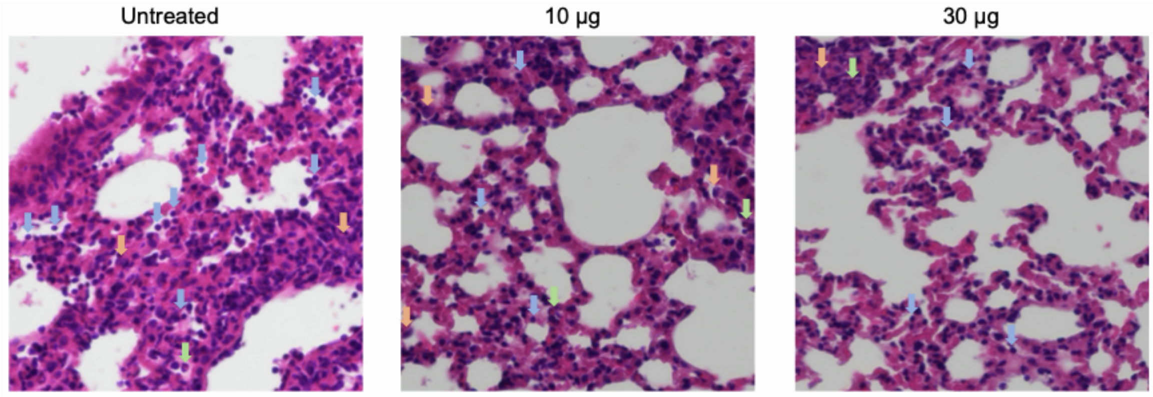

Figure S5. Types of infiltrating immune cells in the lung after SARS-CoV-2 challenges. Magnifications of Figure 2F. Typical images of lymphocytes (blue arrows), eosinophils (green arrows), and histocytes (orange arrows) are highlighted.

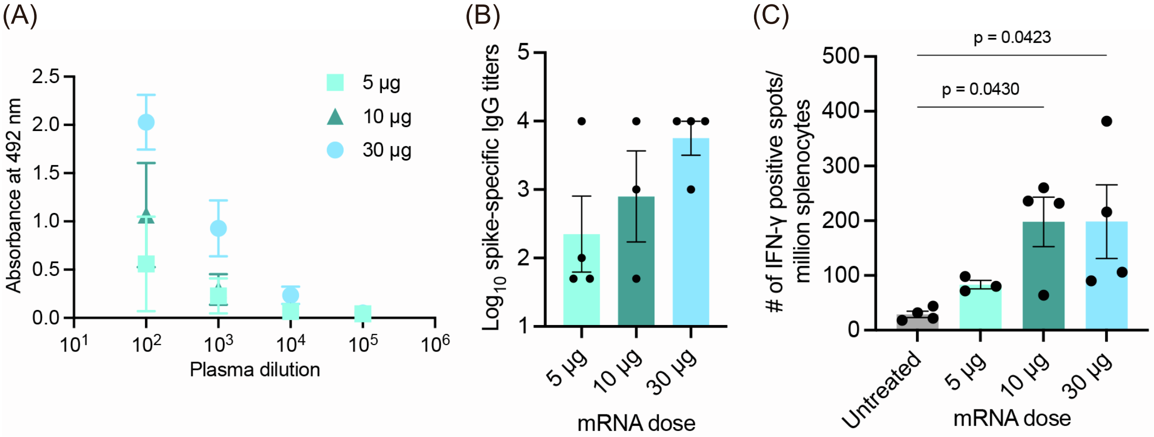

Figure S6. Humoral and cellular immunity of naked *spike* mRNA injected using PYRO in C57BL/6J mice. Mice were injected in a prime-boost setting at a 3-week interval. 2 weeks after the boost, blood plasma and splenocytes were collected for evaluating vaccination effects. (A) Anti-spike IgG ELISA absorbance vs. plasma dilution curves. (B) Log-transformed IgG titers. (C) Quantification of spike-specific IFNγ-positive splenocytes. Data represent the mean ± SEM (n=4). One way ANOVA followed by Dunnett's test.

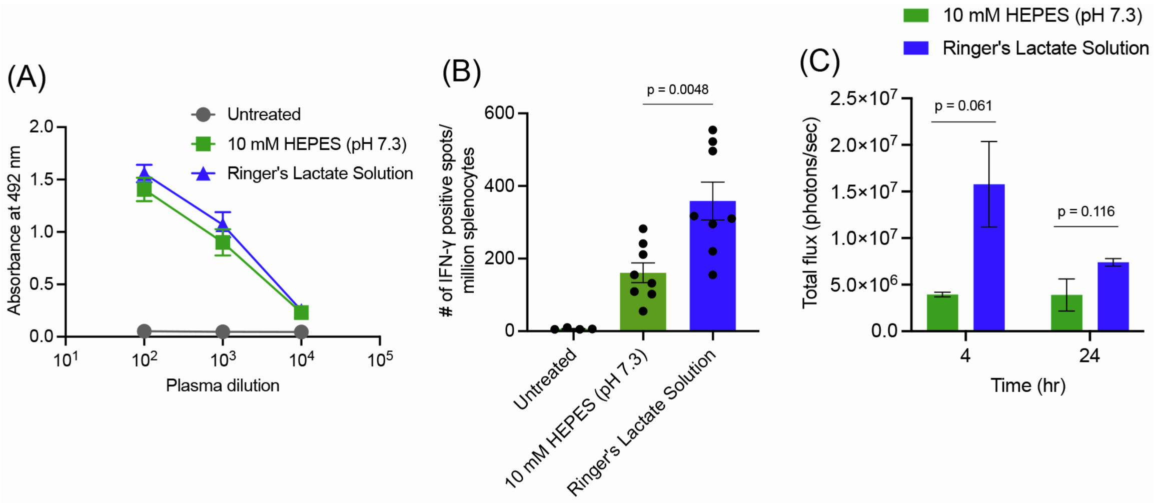

Figure S7. The impact of buffer types on vaccination efficiency following PYRO injection of naked mRNA. 10 µg of *spike* mRNA, dissolved in either 10 mM HEPES buffer or isotonic Ringer's lactate solution, was PYRO-injected into BALB/c mice (female) twice at a 3-week interval. Immunological analyses were conducted 2 weeks after the boost. (A) Spike-specific IgG ELISA absorbance at 100× – 10,000× serum dilution. n = 8. (B) ELISpot for spike-reactive IFN-γ splenocytes. n = 8. (C) fLuc expression efficiency after PYRO-injection of naked *fLuc* mRNA dissolved in either 10 mM HEPES buffer or isotonic Ringer's lactate solution. Luminescence was quantified using IVIS. n = 3. Data represent the mean ± SEM. Statistical analyses were performed by two-tailed Student's t test.

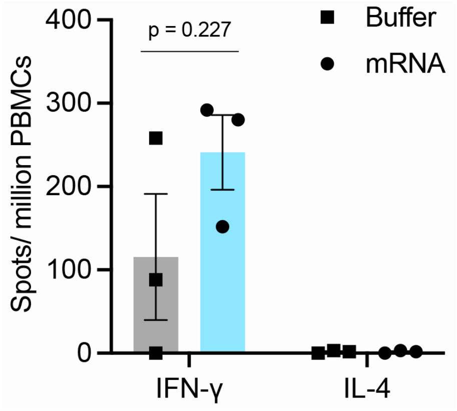

Figure S8. Cellular immunity of naked spike mRNA injected using PYRO in Cynomolgus Monkeys. ELISpot of PBMCs was performed at day 55. Data represent the mean ± SEM (n=3).

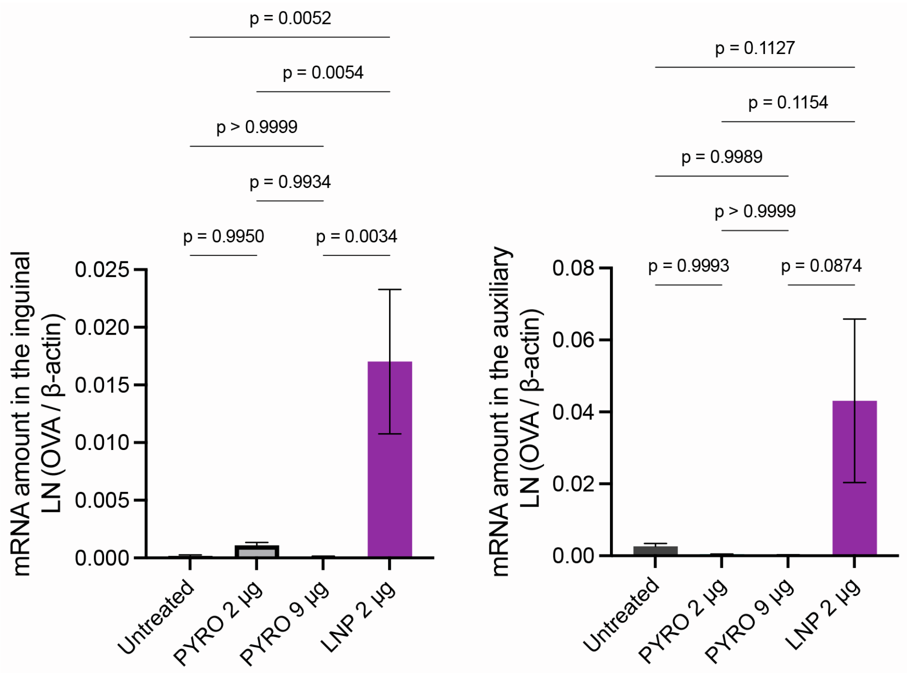

Figure S9. Tissue distribution of injected mRNA. Quantitative PCR (qPCR) of *OVA* mRNA was conducted 30 min after PYRO-injection of naked mRNA or intradermal N&S injection of LNP. Data are normalized to *β-actin* mRNA levels. Data represent the mean ± SEM (n=5-6). $^{**}p<0.01$, $^{*}p<0.05$, non-repeated ANOVA followed by Bonferroni test.

(A)

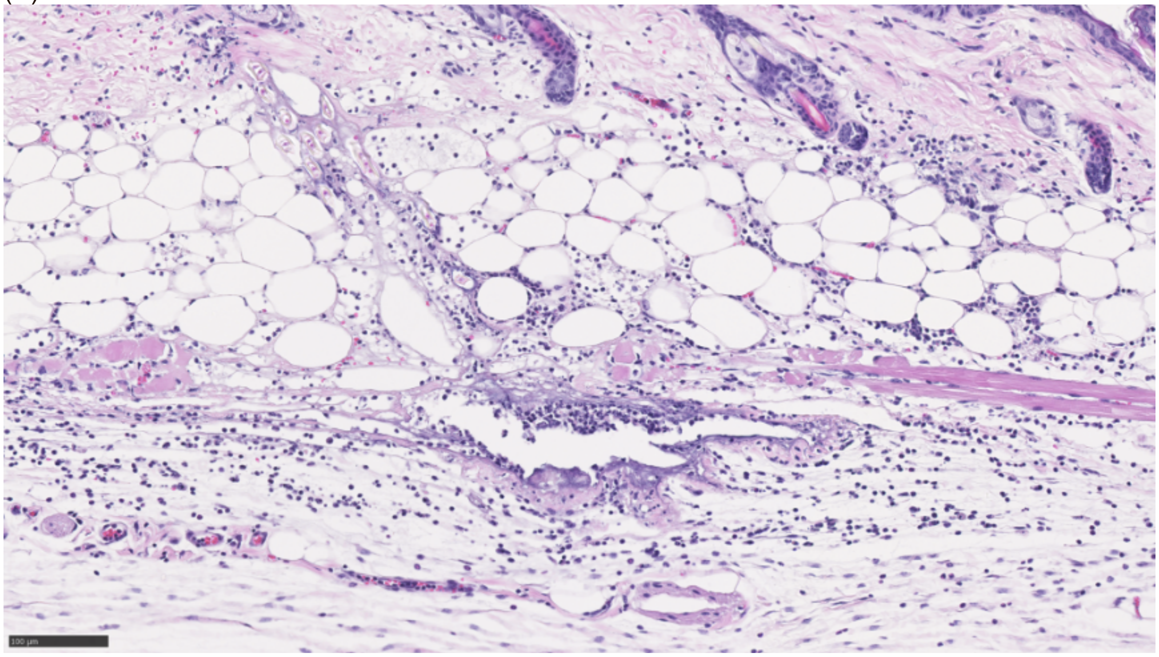

(B)

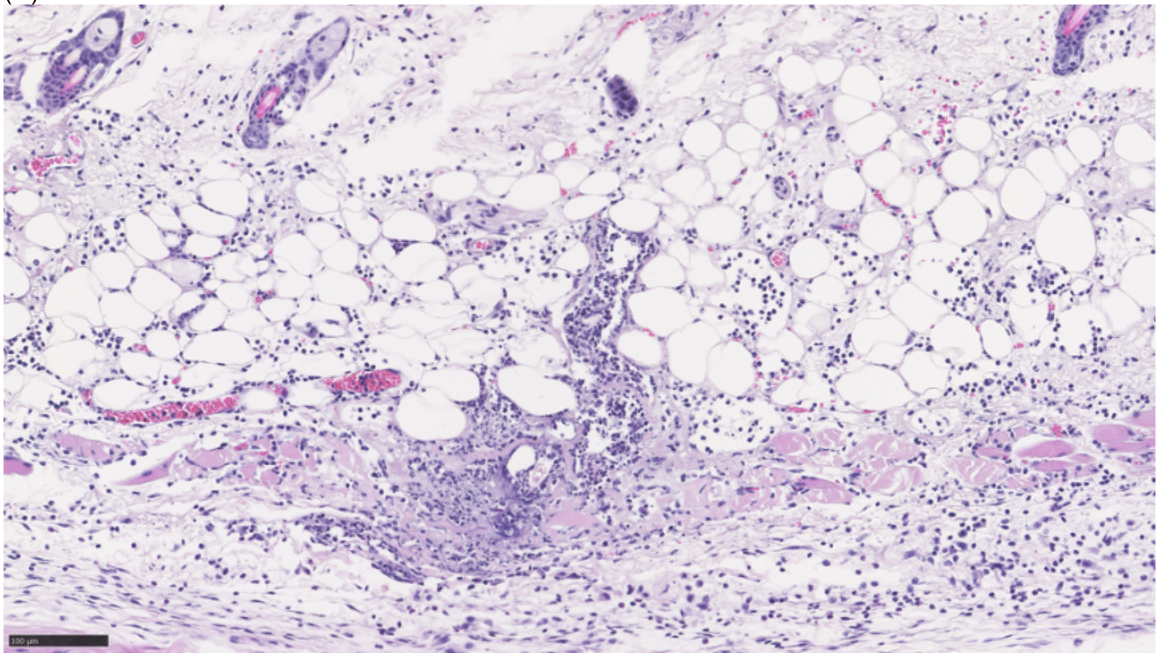

Figure S10. Magnified images of the skin 1 d post PYRO-injection. PYRO injection of (A) mRNA solution and (B) buffer. (A and B) are magnified images of Figures 6 (A and B), respectively. Scale bars: 100 µm.

(A)

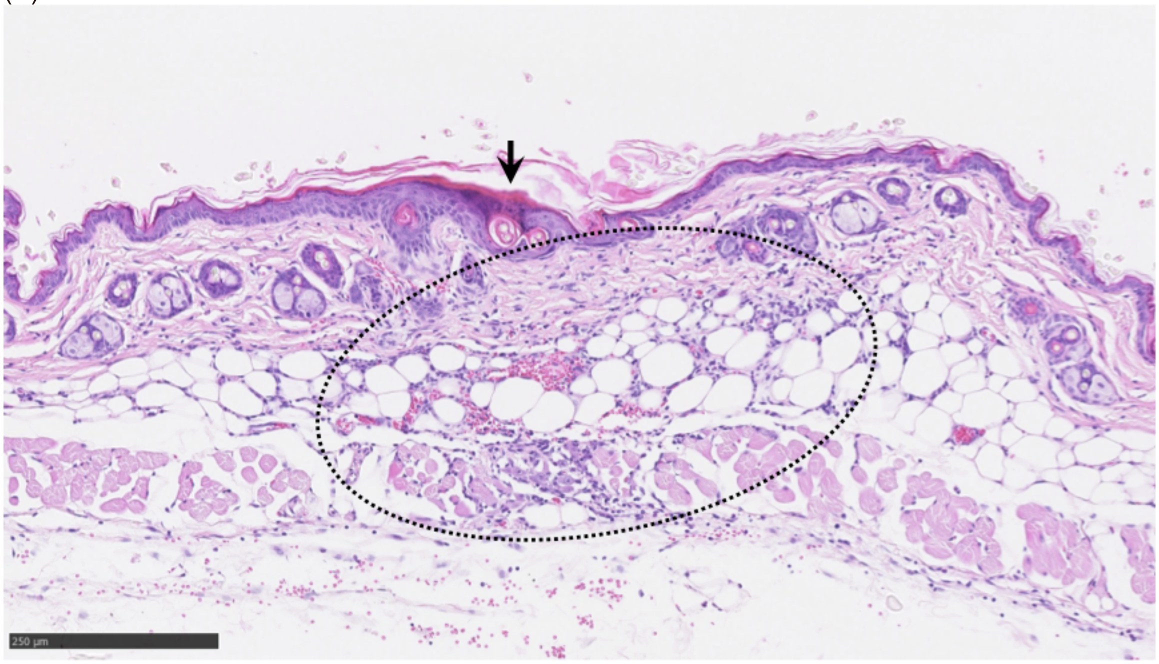

(B)

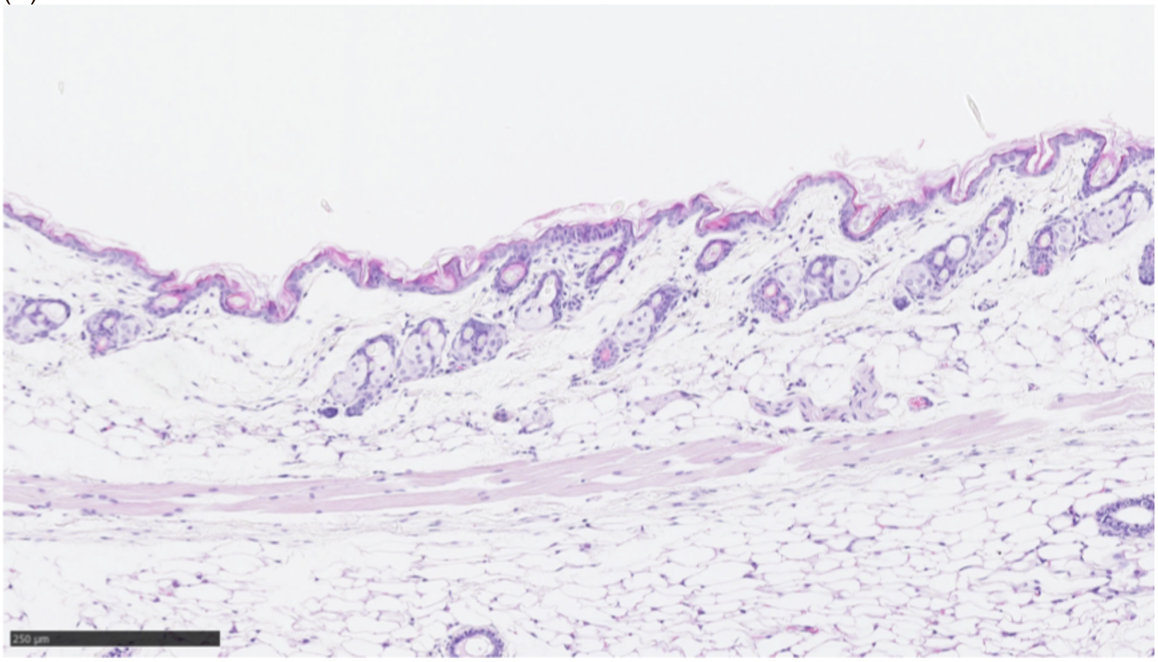

Figure S11. Time-dependent observation of the skin following PYRO injection. H&E staining of the injection site was performed 3 d (A) and 7 d (B) post-injection of mRNA solution. The arrow in (A) indicates the injection site. The dashed circle in (A) shows modest inflammation with immune cell infiltration. Scale bars: 250 μm

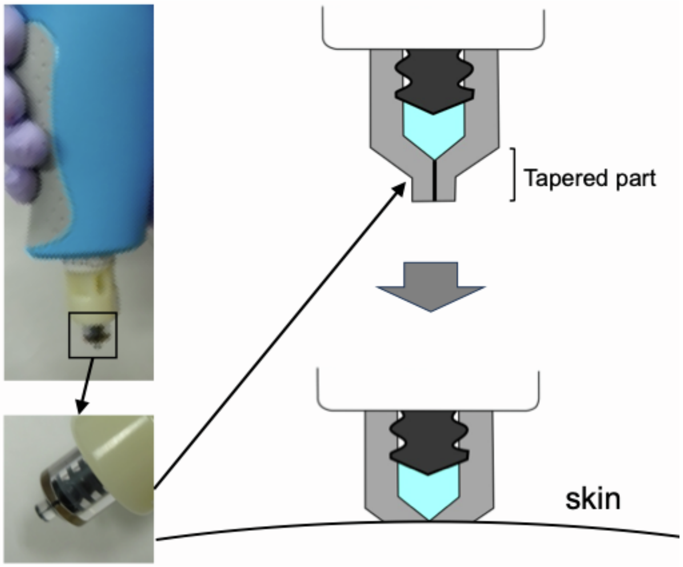

Figure S12. Application of the jet injector to mouse skin. In left pictures, the injector was filled with black ink to visualized the solution.

Table S1. The sizes of the blebs following N&S and PYRO injection. The sizes were measured by a caliper. Data represent the mean ± SEM (n=3).

	Long axis (mm)	Short axis (mm)	Height (mm)
N&S injection	7.49 ± 0.41	5.66 ± 0.12	1.43 ± 0.15
PYRO injection	3.24 ± 0.14	2.75 ± 0.26	0.45 ± 0.06

Table S2. Hematology markers following repeated intradermal injections in Cynomolgus Monkeys using PYRO. Data represent the mean ± SEM (n=3).

	**Injection**	**Day -7**	**Day 20**	**Day 41**	**Day 55**
RBC (10^6/μL)	Buffer	5.23 ± 0.23	5.16 ± 0.09	5.02 ± 0.19	5.04 ± 0.06
	mRNA	5.51 ± 0.22	5.34 ± 0.11	5.08 ± 0.19	5.06 ± 0.09
HGB (g/dL)	Buffer	13.07 ± 0.41	12.80 ± 0.10	12.43 ± 0.35	12.43 ± 0.15
	mRNA	14.13 ± 0.44	13.60 ± 0.17	13.03 ± 0.42	13.00 ± 0.21
HCT (%)	Buffer	42.87 ± 1.60	42.27 ± .72	41.67 ± 1.68	40.93 ± 0.61
	mRNA	45.03 ± 1.57	43.67 ± 0.64	42.47 ± 1.69	42.20 ± 0.74
MCV (fL)	Buffer	25.03 ± 0.41	24.80 ± 0.38	24.80 ± 0.40	24.63 ± 0.38
	mRNA	25.70 ± 0.67	25.50 ± 0.64	25.73 ± 0.65	25.73 ± 0.82
MCH (pg)	Buffer	82.00 ± 0.53	81.87 ± .09	83.03 ± 0.70	81.13 ± 0.37
	mRNA	82.00 ± 1.80	81.87 ± 1.05	83.67 ± 0.91	83.47 ± 2.12
MCHC (g/dL)	Buffer	30.50 ± 0.29	30.30 ± 0.44	29.87 ± 0.38	30.40 ± 0.47
	mRNA	31.40 ± 0.19	31.17 ± 0.44	30.70 ± 0.53	30.83 ± 0.37
RET (%)	Buffer	1.19 ± 0.20	1.19 ± 0.19	1.73 ± .44	1.42 ± 0.26
	mRNA	1.28 ± 0.21	1.86 ± 0.36	2.29 ± 0.36	1.67 ± 0.29
RET (10^9/L)	Buffer	61.17 ± 8.15	61.10 ± 8.98	85.40 ± 19.87	71.33 ± 12.55
	mRNA	70.33 ± 8.71	98.17 ± 16.98	116.17 ± 15.50	84.33 ± 13.52
PLT (10^3/μL)	Buffer	400.0 ± 54.0	376.3 ± 62.4	395.33 ± 63.3	391.7 ± 46.3
	mRNA	321.7 ± 49.3	292.0 ± 58.6	313.3 ± 62.2	324.0 ± 46.3
WBC (10^3/μL)	Buffer	11.47 ± 0.37	13.52 ± 1.54	10.92 ± 0.79	9.69 ± 0.27
	mRNA	8.06 ± 0.67	11.77 ± 1.22	10.08 ± 0.75	9.97 ± 1.07
NEUT (%)	Buffer	28.07 ± 5.17	39.53 ± 7.34	35.53 ± 1.49	36.63 ± 4.78
	mRNA	34.17 ± 2.68	47.90 ± 8.42	40.00 ± 1.72	47.57 ± 8.66
LYMPH (%)	Buffer	68.37 ± 4.83	56.50 ± 6.54	60.60 ± 1.14	59.83 ± 3.82
	mRNA	61.13 ± 3.20	48.33 ± 7.77	55.00 ± 1.58	48.80 ± 8.12
MONO (%)	Buffer	2.87 ± 0.33	3.30 ± 0.87	3.33 ± 0.44	2.97 ± 0.87
	mRNA	3.97 ± 0.64	3.33 ± 0.85	4.30 ± 0.50	3.13 ± 0.81
EO (%)	Buffer	0.57 ± 0.09	0.53 ± 0.15	0.40 ± 0.10	0.47 ± 0.15
	mRNA	0.57 ± 0.06	0.33 ± 0.17	0.50 ± 0.10	0.30 ± 0.18
BASO (%)	Buffer	0.13 ± 0.03	0.13 ± 0.03	0.13 ± 0.03	0.10 ± 0.00
	mRNA	0.17 ± 0.03	0.10 ± 0.00	0.20 ± 0.00	0.20 ± 0.00
PT (s)	Buffer	9.60 ± 0.06	9.43 ± 0.03	9.40 ± 0.06	9.60 ± 0.06
	mRNA	9.90 ± 0.12	9.97 ± 0.06	9.83 ± 0.12	10.20 ± 0.12
APTT (s)	Buffer	23.27 ± 0.43	23.40 ± 0.35	24.03 ± 0.78	23.67 ± 0.50
	mRNA	21.60 ± 1.00	21.77 ± 1.06	21.80 ± 1.33	22.13 ± 1.07

Table S3. Blood chemistry following repeated intradermal injections in Cynomolgus Monkeys using PYRO. Data represent the mean ± SEM (n=3).

	**Injection**	**Day -7**	**Day 20**	**Day 41**	**Day 55**
AST (U/L)	Buffer	39.33 ± 7.84	40.67 ± 11.72	31.33 ± 2.91	28.67 ± 2.33
	mRNA	28.00 ± 10.33	25.33 ± 13.69	24.33 ± 5.46	23.33 ± 4.62
ALT (U/L)	Buffer	57.33 ± 19.41	58.33 ± 19.01	49.00 ± 17.69	56.67 ± 22.93
	mRNA	45.67 ± 21.79	43.67 ± 20.99	35.00 ± 17.09	34.67 ± 23.79
LD (U/L)	Buffer	343.7 ± 41.7	318.3 ± 32.3	294.3 ± 23.3	280.3 ± 12.9
	mRNA	381.0 ± 15.0	345.0 ± 17.5	317.0 ± 22.4	327.0 ± 12.7
CK (U/L)	Buffer	143.7 ± 11.6	157.3 ± 9.0	206.7 ± 74.7	151.3 ± 26.8
	mRNA	151.7 ± 16.7	156.3 ± 22.1	150.3 ± 82.3	137.0 ± 15.0
GLU (mg/dL)	Buffer	104.7 ± 5.6	123.3 ± 25.9	97.0 ± 13.6	71.7 ± 7.4
	mRNA	97.3 ± 4.3	107.7 ± 27.0	137.7 ± 13.4	95.3 ± 1.2
BIL (mg/dL)	Buffer	0.12 ± 0.01	0.11 ± 0.01	0.17 ± 0.02	0.12 ± 0.03
	mRNA	0.11 ± 0.01	0.09 ± 0.01	0.12 ± 0.02	0.09 ± 0.01
UN (mg/dL)	Buffer	20.10 ± 1.39	21.10 ± 1.31	18.73 ± 0.90	20.20 ± 1.10
	mRNA	17.93 ± 1.77	18.47 ± 2.14	16.90 ± 1.76	18.60 ± 1.66
CRE (mg/dL)	Buffer	0.70 ± 0.03	0.70 ± 0.08	0.67 ± 0.08	0.76 ± 0.09
	mRNA	0.67 ± 0.03	0.71 ± 0.04	0.65 ± 0.05	0.68 ± 0.05
CHO (mg/dL)	Buffer	118.7 ± 6.1	119.0 ± 7.6	119.7 ± 6.9	109.3 ± 10.7
	mRNA	131.7 ± 6.4	113.3 ± 8.7	118.0 ± 7.4	111.0 ± 12.5
TG (mg/dL)	Buffer	44.00 ± 0.58	47.00 ± 5.29	34.67 ± 6.33	45.00 ± 8.96
	mRNA	23.67 ± 6.03	21.00 ± 9.82	24.33 ± 6.44	24.67 ± 7.62
PL (mg/dL)	Buffer	200.3 ± 7.2	201.0 ± 4.6	185.0 ± 6.7	189.3 ± 17.8
	mRNA	185.3 ± 11.0	159.7 ± 16.2	160.3 ± 10.1	166.3 ± 17.8
IP (mg/dL)	Buffer	4.17 ± 0.31	3.55 ± 0.58	4.89 ± 0.48	3.74 ± 0.40
	mRNA	4.68 ± 0.25	4.97 ± 0.61	5.20 ± 0.47	4.42 ± 0.18
CA (mg/dL)	Buffer	9.57 ± 0.25	9.34 ± 0.32	9.38 ± 0.32	9.39 ± 0.34
	mRNA	9.35 ± 0.34	9.24 ± 0.30	9.51 ± 0.31	9.28 ± 0.24
NA (mEq/L)	Buffer	149.9 ± 1.0	147.4 ± 1.4	147.7 ± 1.1	149.3 ± 1.9
	mRNA	149.9 ± 1.2	148.8 ± 1.1	149.3 ± 1.1	149.5 ± 1.5
K (mEq/L)	Buffer	4.16 ± 0.10	3.74 ± 0.15	3.88 ± 0.16	3.81 ± 0.08
	mRNA	4.27 ± 0.08	4.15 ± 0.18	4.13 ± 0.14	3.99 ± 0.08
CL (mEq/L)	Buffer	107.5 ± 0.4	105.1 ± 1.3	107.8 ± 1.0	108.5 ± 1.1
	mRNA	112.8 ± 1.7	109.9 ± 3.1	111.8 ± 2.5	111.1 ± 1.3
TP (g/dL)	Buffer	7.33 ± 0.05	7.09 ± 0.07	7.15 ± 0.04	7.09 ± 0.11
	mRNA	6.93 ± 0.24	6.56 ± 0.30	6.56 ± 0.21	6.61 ± 0.22
ALB (g/dL)	Buffer	4.17 ± 0.02	3.99 ± 0.10	4.03 ± 0.09	4.06 ± 0.14
	mRNA	4.06 ± 0.02	3.90 ± 0.11	3.90 ± 0.06	4.03 ± 0.11
A/G	Buffer	1.32 ± 0.01	1.29 ± 0.05	1.30 ± 0.07	1.34 ± 0.06
	mRNA	1.43 ± 0.11	1.48 ± 0.10	1.46 ± 0.06	1.57 ± 0.11

Table S4. *OVA* mRNA-LNP characterization

Size (nm)	Polydispersity index (PDI)	ζ-potential (mV)	Encapsulation efficiency (%)
67	0.13	0.21	92.5
